# Application of change-point analysis to determine winter sleep patterns of the raccoon dog (*Nyctereutes procyonoides*) from body temperature recordings and a multi-faceted dietary and behavioral study of wintering

**DOI:** 10.1186/1472-6785-12-27

**Published:** 2012-12-13

**Authors:** Anne-Mari Mustonen, Terttu Lempiäinen, Mikko Aspelund, Paavo Hellstedt, Katri Ikonen, Juhani Itämies, Ville Vähä, Jaakko Erkinaro, Juha Asikainen, Mervi Kunnasranta, Pekka Niemelä, Jari Aho, Petteri Nieminen

**Affiliations:** 1 Institute of Biomedicine/Anatomy, School of Medicine, Faculty of Health Sciences, University of Eastern Finland, P.O. Box 1627, FI-70211, Kuopio, Finland; 2 Department of Biology, Faculty of Science and Forestry, University of Eastern Finland, P.O. Box 111, FI-80101, Joensuu, Finland; 3 Botanical museum, Department of Biology, University of Turku, FI-20014, Turku, Finland; 4 Department of Physics and Mathematics, Faculty of Science and Forestry, University of Eastern Finland, P.O. Box 111 FI-80101, Joensuu, Finland; 5 Department of Biological and Environmental Sciences, University of Helsinki, P.O. Box 27, FI-00014, Helsinki, Finland; 6 Zoological museum, Department of Biology, University of Oulu, P.O. Box 3000, FI-90014, Oulu, Finland; 7 Finnish Game and Fisheries Research Institute, P.O. Box 413, FI-90014, Oulu, Finland; 8 Finnish Game and Fisheries Research Institute, Itäinen Pitkäkatu 3, FI-20520, Turku, Finland; 9 Department of Biology, University of Turku, FI-20014, Turku, Finland; 10 Municipal Veterinary Clinic of Joensuu, Takilatie 5, FI-80110, Joensuu, Finland

**Keywords:** Body temperature, Change-point analysis, Fatty acid signature, Foraging ecology, GPS tracking, Home range, *Nyctereutes procyonoides*, Winter sleep

## Abstract

**Background:**

A multi-faceted approach was used to investigate the wintertime ecophysiology and behavioral patterns of the raccoon dog, *Nyctereutes procyonoides*, a suitable model for winter sleep studies. By utilizing GPS tracking, activity sensors, body temperature (T_b_) recordings, change-point analysis (CPA), home range, habitat and dietary analyses, as well as fatty acid signatures (FAS), the impact of the species on wintertime food webs was assessed. The timing of passive bouts was determined with multiple methods and compared to T_b_ data analyzed by CPA.

**Results:**

Raccoon dogs displayed wintertime mobility, and the home range sizes determined by GPS were similar or larger than previous estimates by radio tracking. The preferred habitats were gardens, shores, deciduous forests, and sparsely forested areas. Fields had close to neutral preference; roads and railroads were utilized as travel routes. Raccoon dogs participated actively in the food web and gained benefit from human activity. Mammals, plants, birds, and discarded fish comprised the most important dietary classes, and the consumption of fish could be detected in FAS. Ambient temperature was an important external factor influencing T_b_ and activity. The timing of passive periods approximated by behavioral data and by CPA shared 91% similarity.

**Conclusions:**

Passive periods can be determined with CPA from T_b_ recordings without the previously used time-consuming and expensive methods. It would be possible to recruit more animals by using the simple methods of data loggers and ear tags. Hunting could be used as a tool to return the ear-tagged individuals allowing the economical extension of follow-up studies. The T_b_ and CPA methods could be applied to other northern carnivores.

## Background

To yield reliable data not influenced by human disturbance, several methods have been developed to monitor activity patterns of wild mammals. Very high frequency (VHF) tracking is most often utilized but it is time-consuming and labour-intensive. This makes the use of radio telemetry expensive, although radio collars are generally quite cheap and their longevity is good. Global positioning system (GPS) tracking yields more abundant and accurate home range data but the equipment is expensive and fails more easily [1]. Although GPS systems cost more than VHF collars, the cost per one location is often smaller, as personnel costs for field work can be minimized.

Monitoring winter activity is essential for describing the behavior of species utilizing a complex wintering strategy consisting of alternating periods of physical activity and passivity. Determining the duration and timing of passive bouts can be useful in several ways. Winter sleep patterns could be used as an indicator for climate change scenarios to monitor the effects of global warming on boreal ecosystems. The climate change can lead to higher foraging effort in winter increasing intra- and interspecies interactions and, thus, also the transmission risk of rabies, other zoonoses, and parasites [2]. For this reason, activity of passively wintering species should be re-evaluated with modern tracking methods.

A very useful model species is the palearctic raccoon dog (*Nyctereutes procyonoides*), an invasive omnivore, which inhabits temperate to subarctic regions. It is very abundant, harvested legally, and due to its relatively small body size, it can be handled safely and, e.g., anesthesia is not needed for procedures such as blood sampling. In Finland, the raccoon dog is considered an alien species and its population size has showed an increasing trend [3]. Recently, its area of distribution has expanded quite rapidly to the northernmost Lapland, and Finnish individuals are also colonizing Sweden via the northern route. The goals of the population control include the prevention of the spread and establishment of the species to other parts of Scandinavia, as it is a vector of diseases and parasites and a potential threat to native fauna. It has also been pondered if the spread of the distribution area of the raccoon dog could benefit from global warming [4]. The ecophysiology of overwintering of the species was previously investigated with traditional methods [5,6]. As the raccoon dog displays an intermediate wintering strategy between bears (*Ursus* spp.) and actively wintering carnivores (e.g., the red fox *Vulpes vulpes*), studying its ecophysiology can increase the knowledge on the evolution of different types of wintering.

For these reasons, it would be desirable to be able to define the resting periods of animals economically without compromising the reliability of the data. The timing of winter sleep bouts was previously approximated from the core body temperature (T_b_) data of raccoon dogs [5]. Due to this, it would be possible to equip animals with inexpensive intra-abdominal temperature loggers and ear tags, and to identify the periods of passive wintering from the T_b_ data of each individual. To be able to perform a more detailed analysis of the patterns of alternating passivity and active foraging bouts, a reliable mathematical application that would be able to detect more short-term and subtle changes in T_b_ rhythms and that would be less dependent on subjective human assessment could be most useful.

The aim of the present set of experiments was to conduct a comprehensive study on several previously neglected aspects of overwintering of the raccoon dog. The species could have a significant impact on food webs during its activity bouts in winter. This can be assessed *i*) by determining the utilization of different biotopes and road/railroad networks by positioning data and habitat use analysis. The wintertime diet of the species can be investigated *ii*) by analyzing the contents of gastrointestinal tracts together with adipose tissue fatty acids (FA) and FA signatures (FAS). *iii*) External factors that change during global warming and affect the timing and duration of passivity can also be determined. Finally, *iv*) a mathematical method analyzing T_b_ patterns can determine the time periods when raccoon dogs operate actively in wintertime food webs. The hypotheses of the study were as follows: *i*) abiotic factors in winter (ambient temperature T_a_, snow) can affect the habitat use of raccoon dogs and direct them to utilize road/railroad networks as pathways for foraging, *ii*) raccoon dogs utilize food resources opportunistically during wintertime activity bouts and can have an impact on food webs in their habitats, *iii*) factors liable to the climate change (T_a_, snow depth) can have an effect on the activity patterns and, thus, winter sleep could be susceptible to global warming, and *iv*) T_b_ patterns can be subjected to mathematical analysis interpreting reliably the passive periods of wintering from the T_b_ data of wild raccoon dogs.

The present study developed a practical tool to assess from easily obtainable data (intra-abdominal T_b_) the periods of passivity, the determination of which would otherwise require either extensive work in the field (radio tracking) or expensive equipment (GPS collars and activity sensors combined with data transfer costs). This novel and economical method utilizing change-point analysis (CPA) can be used not only in research projects on the raccoon dog but eventually applied also to other passively wintering carnivores.

## Methods

### Body temperature, activity, and home ranges

The study procedures were approved by the Animal Care and Use Committee of the University of Joensuu in 2006–2007 and by the Finnish National Animal Experiment Board in 2008–2010 (#ESLH-2008-06316/Ym-23), and complied with the current laws of Finland. Fourteen raccoon dogs were captured live with box traps, dogs, or using a cable in Kaatamo and Ristinkylä villages in eastern Finland (62.56116338 N; 29.14269194 E) in autumn 2006 and 2007. They were anesthetized with intramuscular ketamine (5 mg/kg) and xylazine (2 mg/kg) and two sterile thermosensitive data loggers (iButton Thermochron DS1921H, Maxim Integrated Products, Sunnyvale, CA) registering the T_b_ at 120-min intervals were implanted into their abdominal cavities [5]. The accuracy and precision of the loggers had been tested rigorously [7]. The animals were fitted with ear tags (model 1841, National Band & Tag Co, Newport, KY) and store-on-board GPS collars with remote GSM and a back-up VHF radio beacon (Tellus Basic collar 2A, Followit AB, Stockholm, Sweden; weight 3–4% of body mass, BM). The GPS collars were programmed to record 8 position coordinates per day (at 01:00, 02:00, 04:00, 10:00, 20:00, 22:00, 23:00, 24:00 h) based on the mostly nocturnal activity pattern of the species [2]. One daytime fix was obtained to position the rest site. The activity sensors measured the change in the acceleration of the collar in two axes (x, y) during each 120-s time period used to obtain a GPS fix. No exact ranges of activity values indicating activity *vs*. passivity were determined previously for the studied species, and for the present experiment, the activity score sum [Σ(x + y)] was calculated for each date of each individual.

BM and body lengths were determined and body mass indices (BMI) correlating with the body fat-% [8] calculated. Age was estimated by palpation of the prominence of the ulna (closure of the epiphyseal plate) and, if a hunted animal was delivered to the University, *post mortem* with histological examination of a canine tooth root [9]. The individuals <1 year of age were considered juveniles and those >1 year of age were classified as adults. A blood sample was taken from a saphenous vein of a hind leg with sterile needles and syringes with ethylenediaminetetraacetic acid, and the complete blood count (CBC) was determined as an indicator of health with the Vet abc Animal Blood Counter (ABX Hematologie, Montpellier, France), adjusted to the canine hematologic profile. A sample (1–2 g) of ventral subcutaneous (sc) fat was removed and stored at –80°C. After a recovery period of approximately one week at a fur farm, the animals were released at the capture sites, recaptured live or dead in the following spring, and the live individuals were operated similarly. Moreover, 6 raccoon dogs were captured, operated, and released uncollared in autumn 2007, 2008, and 2009. They were returned dead by hunters in the subsequent springs, and the data loggers were recovered. Taken together, T_b_ loggers were retrieved from 10 collared animals [2 adult males (M1, M3), 3 juvenile males (M2, M5, M6), 3 adult females (F3, F4, F6), 2 juvenile females (F1, F2)] and 6 uncollared animals [1 adult male (M9), 1 juvenile male (M8), 1 adult female (F11), 3 juvenile females (F8, F9, F10)]. GPS collars were recovered from 12 individuals [3 adult males (M1, M3, M4), 3 juvenile males (M2, M5, M6), 3 adult females (F3, F4, F6), 3 juvenile females (F1, F2, F5)], as two collars got dysfunctional.

All successive relocations were included in the home range analyses although they may have been autocorrelated, i.e., taken too closely in time to be statistically independent, to get the best available estimates [10-12]. The fixed Kernel method (K95/50%; [13]) was used to estimate the home range sizes and core areas (50% of the locations) together with the incremental analysis using the Ranges7 software (Anatrack Ltd, Wareham, UK; [14]). Two juveniles performed long-distance dispersion from one home range to another. For F2, the relocations of these home ranges were analyzed separately, and for M5, only the postdispersal relocations were included in the home range analysis as, based on the incremental analysis and previous data [15], the number of fixes in the initial home range was insufficient. The calculated total home range areas were plotted on a habitat map (CLC2000, European Environment Agency, Copenhagen, Denmark) in a geographic information system (ArcGIS 10, Esri, Redlands, CA) to assess the relative importance of each habitat type during winter. Habitat selection was analyzed by determining, firstly, which habitats were used based on the relocations of the individuals. Secondly, this was compared to the overall habitat distribution in the home range. A selection index [(% of relocations)/(% of potentially available habitat)] was calculated for each habitat type to determine, which habitats were favored or ranked low.

The wintertime T_b_ recordings (principally in Nov–March) were divided into active and passive periods based on the ability of the collar to position the animal, relocations, and activity scores. The (x + y)-values of the activity sensors were evaluated together with the positioning data to assess activity/passivity (activity scores + positioning = behavioral data). An animal was considered passive when it stayed at the same GPS position, its collar could not establish satellite connection (i.e., the animal was most likely below the ground or hiding in dense undergrowth vegetation), and/or when its 24-h activity scores were close to zero or, based on the general appearance of individual activity data, lower than during the nights with documented foraging. Snow depth was monitored once a week and after each snowfall, and thermosensitive loggers (iButton Thermochron DS1921G) synchronized with the T_b_ recordings registered the T_a_ at 2-h intervals.

### Dietary analyses and fatty acid signatures

The composition of the diet was studied from 93 fresh carcasses harvested during the periods of snow cover between Nov 5 2006–April 13 2007 and Nov 9 2007–March 30 2008. Carcasses were sexed and weighed, and the thickness of the ventral sc fat and the mass of the omentum were measured. The gastrointestinal tract was dissected, weighed, and frozen at –20°C. If the animal had been killed <24 hrs earlier and stored frozen, 1–2 g of ventral sc fat was dissected and stored at –80°C for FAS analysis.

After thawing, the stomachs and intestines were separated, weighed full, opened, rinsed in a sieve (mesh size 0.5 mm), and weighed empty. Their contents were stored in EtOH. Experts identified the undigested food remains (teeth, bones, hairs, feathers, seeds, etc.) macro- and microscopically to the lowest possible taxon [16-19]. No microscopic search was performed for earthworm chetae as ground frost presumably made the access to earthworms very unlikely, and their importance was previously negligible in winter diet [20]. Hair samples were laid on a strip of balsa wood and fixed using colourless nail varnish. Dried samples were cut with a razor blade, the cross-section was viewed microscopically, and the species/genus identified [21,22]. The volumes of food remains were measured using glass measuring cylinders. The food items were classified into 13 categories: cereals, berries, vegetables, fruits, rodents, insectivores, carnivores, leporids, cervids, birds, fish, invertebrates, and other digestible material that could not be further classified. The total of useful plants was calculated as the sum of agricultural and utility plants. Likewise, sums of wild, digestible, and undigestible plants, and small, medium-sized, and large mammals were calculated.

The results are presented as the volume (ml) of each food item in the stomachs/intestines and as the volume of each food item of the total volume of the stomach/intestinal food items (relative share, RS%). Man-made material, undigestible plant material, soil, and small amounts of raccoon dog hairs presumably ingested during grooming were excluded, as were the ingested baits from the traps. Vol1 stands for the total volume of all digestible food items excluding baits, whereas Vol2 signifies the total volume of all ingested material including nondigestible constituents, baits, and groomed raccoon dog hair. The frequency of occurrence (FO) was calculated as follows: *i*) FO1 = 100 × the proportion of stomachs/intestines containing each food item and *ii*) FO2 = 100 × the occurrence of each food item/the total number of occurrences of all food items. The diet diversity (number of different food items per stomach/intestine) and the main food (the most voluminous food type per stomach/intestine) were also determined. Digestive tract contents were examined macroscopically for roundworms.

The FA composition reflecting the more long-term dietary habits was determined from the sc fat of T_b_ logger-implanted raccoon dogs in 2004–2008 (n = 52; [5,23]) and of freshly frozen carcasses in 2006–2008 (n = 33). The samples were transmethylated by heating with 1% H_2_SO_4_ in methanol under nitrogen atmosphere, the FA methyl esters were extracted with hexane and analyzed by a gas–liquid chromatograph (6890N; Agilent Technologies Inc, Santa Clara, CA) as described previously [23]. Relative changes in the proportions of FA during wintering were calculated by the formula [(mol-% in spring)–(mol-% in autumn)]/[mol-% in autumn]. These were calculated for the individuals that could be captured both in autumn and subsequent spring (n = 14), while the seasonal FA profiles were determined from all animals (autumn n = 39, spring n = 46).

### Change-point analysis of body temperatures

The time periods of passivity determined by behavioral data were compared to passive periods defined from T_b_ data by CPA. It is a powerful tool for detecting changes in a time-series. A simple procedure for performing CPA was introduced by Taylor [24] based on the assumption of the mean-shift data. *X*_1_, *X*_2_, … represent the data in time-order. The mean-shift model can be written as *X*_*i*_ = *μ*_*i*_ + *ε*_*i*_, where *μ*_*i*_ is the average at time *i* and *ε*_*i*_ is the random error associated with the *i*^th^ value. Generally *μ*_*i*_ = *μ*_*i* − 1_ except for a small number of values of *i* called the change-points. It is assumed that random error terms *ε*_*i*_ are independent and their means are zero. The pattern test was utilized for recognizing the mean-shift data [25].

The procedure for performing CPA uses a combination of cumulative sum (CUSUM) charts and bootstrapping to detect the changes. CUSUM charts are constructed by calculating a CUSUM based on the data. These sums are the CUSUM of the differences between the values and the average. When *X*_1_, *X*_2_, …, *X*_*n*_ represent the data, the CUSUM are calculated as follows: 1) calculating the average of the data $\bar{X}$, 2) starting the CUSUM at zero by setting *S*_0_ = 0, and 3) calculating the other CUSUM by adding the difference between the current value and the average to the previous sum: $S_{t}=S_{t-1}+\left(X_{t}-\bar{X}\right)$ for *t* = 1, …, *n*. These CUSUM values produce a time-series *S*_0_, …, *S*_*n*_. The graph of this series (CUSUM chart) can be used for interpreting the occurrence of one or more changes. A segment of the CUSUM chart with an upward slope indicates a period where the values tend to be above the overall average and *vice versa*. A sudden change in the direction of the CUSUM series indicates a sudden shift in the average.

A confidence level for a possible change can be performed by a bootstrap analysis, for which an estimator of the magnitude of the change is required. We used *S*_*diff*_, defined as *S*_*diff*_ = *S*_max_ − *S*_min_, where *S*_max_ is the maximum and *S*_min_ the minimum of the CUSUM series. A single bootstrap analysis can be performed by 1) generating a bootstrap sample, denoted *X*_1_^0^, … *X*_*n*_^0^, by randomly reordering the original values *X*_1_, …, *X*_*n*_, 2) calculating the bootstrap CUSUM based on the bootstrap sample, denoted *S*_0_^0^, …, *S*_*n*_^0^, 3) calculating the *S*_*diff*_^0^, and 4) determining, whether the bootstrap difference *S*_*diff*_^0^ is less than the original difference *S*_*diff*_.

The bootstrap samples represent the case where changes have not occurred. By performing a large number of bootstrap samples and comparing the estimators *S*_*diff*_^0^ with *S*_*diff*_, it can be determined, at which probability at least one change has occurred. The confidence level is calculated as 100 × *X/N*%, where *N* is the number of bootstrap samples and *X* is the number of bootstraps for which *S*_*diff*_^0^ <*S*_*diff*_. A 95% confidence level is required for determining that a change has occurred. Once a change has been detected, the timing of the change can be estimated with the mean square error (MSE) estimator. The MSE(*m*) is defined as:

$$\begin{array}{l} \mathrm{MSE}\left(m\right)=\sum_{i=1}^{m}{\left(X_{i}-{\bar{X}}_{1}\right)}^{2}+\sum_{i=m+1}^{n}{\left(X_{i}-{\bar{X}}_{2}\right)}^{2},\\ \text{where}\;{\bar{X}}_{1}=\frac{\sum_{i=1}^{m}X_{i}}{m}\text{and}\;{\bar{X}}_{2}=\frac{\sum_{i=m+1}^{n}X_{i}}{n-m}. \end{array}$$

The MSE estimator is based on the splitting of the data into two segments, 1 to *m* and *m* + 1 to *n*, estimating the average of each segment and checking how well the data fit the two estimated averages. The value of *m* that minimizes the MSE(*m*) is the best estimator of the last point before the change, and *m* + 1 estimates the first point after the change. Once a change has been detected, the data can be divided into two segments, one on each side of the change-point. Then the analysis is repeated for each segment. For each significant change found, the segments continue to be split in two, etc., to be able to detect multiple changes.

**Figure 1:**
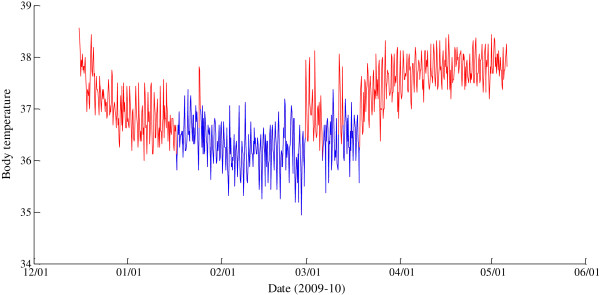
**Passive periods of wintering determined by change-point analysis from body temperature data.** The body temperature (°C) of an adult male raccoon dog (M9) was measured every 120 min with intra-abdominal data loggers in winter 2009–2010, red line = active periods, blue line = passive wintering.

CPA was applied to the T_b_ data for detecting the periods of passivity by separating the data into distinct segments. The original data did not necessarily satisfy the mean-shift assumption as consecutive values may have been correlated. This correlation could be eliminated by handling the series of averages of two consecutive values. The transformed data still included a periodic structure caused by the 24-h rhythm of T_b_, and for this reason, there was a conflict with the assumption of independent error structure. To circumvent this problem, the data were separated into short overlapping segments, as when the segments are short enough, there is no strong evidence of periodicity in each segment. By applying CPA to these segments, the whole original data could be divided into segments with different averages. The periods with the lowest averages and the periods with relatively low averages surrounded by periods with higher averages were most probably the passive periods. The CPA application was developed by using the MATLAB program (*v*R2008a, MathWorks, Natick, MA). An example of the output can be examined in Figure 1 representing the original T_b_ data of M9.

### Statistical analyses

Differences in the general variables between the months were analyzed with the one-way analysis of variance (ANOVA) or the nonparametric Kruskal–Wallis ANOVA (SPSS *v*16.0 software package, SPSS Inc, Chicago, IL). The homogeneity of variances and normality of distribution were tested with the Levene’s and Kolmogorov–Smirnov tests, respectively. The activity score sum and average 24-h T_b_ were calculated for each date of each raccoon dog, and individual amplitude spectra were calculated with the Fast Fourier Transform. The values during active and passive wintering were compared with the independent samples Student’s *t*-test or Mann–Whitney *U*-test for parametric and nonparametric data, respectively. The latter was used when normality of distribution was not attained. The occurrence of food items in the gastrointestinal tracts was tested with the *χ*^2^-test. To analyze the temporal changes in the diet, winter was divided into Nov–Dec (fat storage completed and periods of passivity begin to occur gradually), Jan–Feb (low foraging activity), and March–April (increasing activity and mating season). To analyze the interrelationships between the average 24-h T_b_ and covariates T_a_, day length, and snow depth, the analysis of covariance (ANCOVA) was performed with the linear mixed model analysis. The model included the individual as a random factor and the covariates T_a_, day length, snow depth, and bearing capacity as well as their interactions with the individual. To analyze the relationships between the FAS and different dietary classes, the data were subjected to the multivariate principal component analysis (PCA) using the SIRIUS *v*6.5 software package (Pattern Recognition Systems AS, Bergen, Norway; [26]). Bivariate correlations were calculated with the Spearman correlation coefficient (r_s_). *P* < 0.05 was considered statistically significant. The results are presented as the mean ± SE.

## Results

### Body temperature patterns and their mathematical analysis

According to CPA, the average number of passive periods was 7 ± 0.6, the sum of passive days was 50 ± 5.4, and the durations of the shortest and longest passive periods were 1 ± 0.02 and 31 ± 7.1 days, respectively, in the GPS collared individuals tracked for 3–5 months in Nov–April (Group 1, n = 7). In these animals, the compatibility of the timing of the passive periods, determined by CPA and by behavioral data, was 91% (78–100%). The average number of passive periods (8 ± 1.2), the sum of passive days (56 ± 4.2), and the durations of the shortest and longest passive periods (3 ± 0.7 and 21 ± 4.2 days) of the uncollared animals (Group 2, n = 5) T_b_-recorded but not tracked during the same time period did not differ from Group 1, except of the duration of the shortest passive period (Kruskal–Wallis ANOVA, *H* = 9.467, df = 2, *p* < 0.01). The animals that could be tracked for only shorter periods of time (1–3 months) during Nov–Feb (killed by hunters, dogs, or traffic; Group 3, n = 4) displayed, on average, 1 ± 0.5 passive periods, the total length of passive wintering was 6 ± 2.4 days, and the shortest and longest periods lasted for 3 ± 0.9 and 7 ± 1.5 days (Kruskal–Wallis ANOVA, *H* = 6.307–9.467, df = 2, *p* < 0.01–0.05 *vs.* Group 1). The compatibility of the timing of the passive periods determined by CPA and by behavioral data varied slightly more than in Group 1 (mean 83%, range 63–100%).

**Figure 2:**
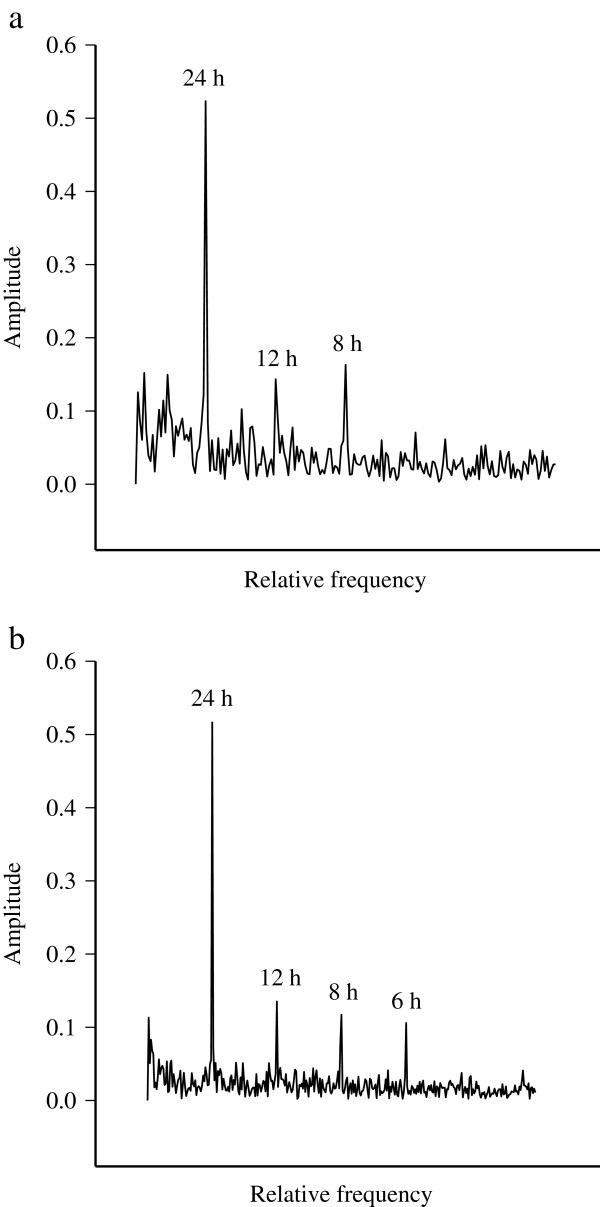
**Representative body temperature amplitude spectra during overwintering.** The body temperature amplitude spectra of an adult male raccoon dog (M1) during **(a)** active and **(b)** passive periods of wintering.

**Figure 3:**
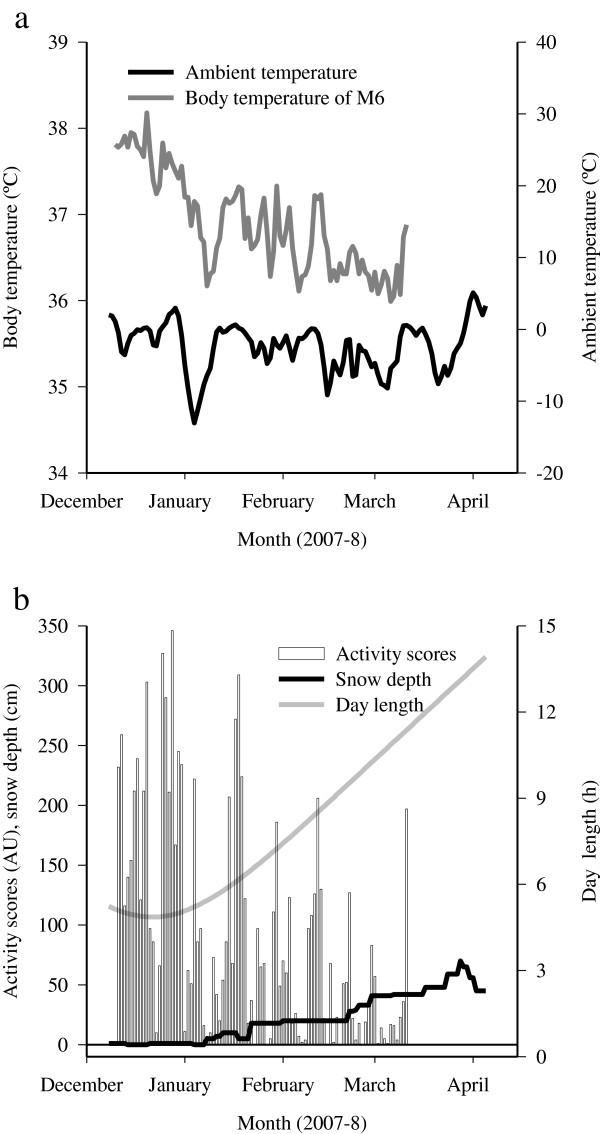
**Average 24-h body temperatures and activity score sums and their relation to external factors. (a)** The average 24-h body temperatures (°C) and **(b)** nocturnal activity score sums (arbitrary units; AU) of a juvenile male raccoon dog (M6) were measured in Dec 2007–March 2008 together with the average 24-h ambient temperatures (°C), day length (h), and snow depth (cm).

Generally, the longest passive period occurred in Jan–March. It was preceded by 1–6 (3.8 ± 0.6) and followed by 0–8 (2.6 ± 0.7) shorter passive periods. The total duration of passivity (days) correlated with the number of passive periods (r_s_ = 0.620, n = 16, *p* < 0.01) and with the duration of the longest passive period (r_s_ = 0.810, n = 15, *p* < 0.001). According to the active/passive classification by CPA, the average T_b_ was 37.4 ± 0.08°C during active and 36.5 ± 0.08°C during passive wintering, the difference being 1.0 ± 0.07°C (Mann–Whitney *U*-test, *U* = 4.000, n = 15, *p* < 0.001). There were no differences in the averages between the study groups. The spectral analysis revealed clear 24-, 12-, and 8-h oscillations in the T_b_ of all animals without significant differences in the magnitude between the active and passive periods (Figure 2). The average 24-h T_b_ was 37.6 ± 0.03°C in Nov–Dec, 36.7 ± 0.03°C in Jan–Feb, and 37.3 ± 0.07°C in March–April, all significantly different from each other (Kruskal–Wallis ANOVA, *H* = 87.716, df = 2, *p* < 0.001). The average 24-h T_b_ showed positive covariance with the average 24-h T_a_ (ANCOVA, *F*_*1,8.001*_ = 19.489, *p* < 0.01), while the interactions with the total depth of snow, depth of soft snow, and day length were nonsignificant (Figure 3). The average 24-h T_b_ of the two individuals wintering together (F8, M8) correlated positively (r_s_ = 0.854, n = 87, *p* < 0.001), and the timing of their passive periods determined by CPA was 89% identical.

### Activity patterns

The average activity score was 86% lower during passive overwintering than during the active periods defined by CPA (*t*-test, |*t*| = 14.259, n = 64, 80, *p* < 0.001). During the active periods of wintering, the average activity scores of specific time points were higher at 20:00–04:00 h than at 10:00 h (ANOVA, *F*_*7,72*_ = 8.667, *p* < 0.001), but during passive wintering the mean values did not differ at any time points. There was a positive correlation between the activity scores and T_b_ of 9/9 individuals when simultaneous activity and T_b_ values were included in the analysis (r_s_ = 0.348–0.611, n = 162–780, *p* < 0.001), and in 7/9 animals when 24-h sums of activity scores and average 24-h T_b_ values were correlated (r_s_ = 0.339–0.767, n = 26–129, *p* < 0.05). The average 24-h activity score had positive covariance with the average 24-h T_a_ (ANCOVA, *F*_*1,6.645*_ = 11.019, *p* < 0.05) and negative covariance with photoperiod (*F*_*1,510.584*_ = 9.288, *p* < 0.01) and depth of snow (*F*_*1,11.384*_ = 5.077, *p* < 0.05; Figure 3), while there was no significant covariance between the activity and depth of soft snow.

### Dietary analysis and fatty acid signatures

The stomachs had no digestible material in 15 (16%) and the intestines in 5 cases (5%). The total mass of the contents varied between 0–987 g. The mass was the highest in Nov (287 ± 48 g), decreased between Nov and Jan, was the lowest in Feb (13 ± 4 g), and increased between Feb and April (Kruskal–Wallis ANOVA, *H* = 34.303, df = 5, *p* < 0.001). The average volumes of all digestible food items were 71 ± 11 ml (stomachs) and 21 ± 3 ml (intestines; see Additional file 1). Undigestible, man-made material (pieces of paper, plastic, cigarette butts, etc.) was found in 27% of the stomachs and 23% of the intestines. Roundworms were present in 29% of the individuals with higher occurrence in Nov–Jan compared to Feb–April (*χ*^2^-test, *χ*^*2*^ = 4.750, df = 1, *p* < 0.05).

Based on the FO1–2, volumes, and RS%, the importance of the main groups of food items decreased as follows: mammals ≥ plants ≥ birds ≥ fish ≥ invertebrates (see Additional file 2, Additional file 3, Additional file 4, Additional file 5, Additional file 6, Additional file 7, Additional file 8, Additional file 9). Twelve mammals could be classified to the species level in the stomachs and 10 in the intestines. Rodents were the most common food items among mammals (see Additional file 2, Additional file 3, and Additional file 9). They consisted mainly of bank voles (*Myodes glareolus*), field voles (*Microtus agrestis*), and unidentified *Microtus* voles, but there were also several observations of red squirrels (*Sciurus vulgaris*). Rodents were accompanied by insectivores (almost exclusively *Sorex* spp. shrews), hares (*Lepus* spp.), carnivores (almost exclusively raccoon dogs), and cervids (Cervidae) with relatively equal occurrences. There were no differences in the FO1–2, volumes, or RS% of these food items between the stomachs and intestines.

Twenty edible plants could be classified to the species level in the stomachs and 18 plants in the intestines (see Additional file 4, Additional file 5, and Additional file 9). Cereals were the most commonly utilized plant-based food followed by berries, vegetables, and fruits. *Avena sativa* was the most important cereal assessed by FO1–2 and volume, and the gastrointestinal tracts of some individuals contained also small volumes of *Panicum miliaceum*. The most common berries were *Sorbus aucuparia*, *Vaccinium vitis-idaea*, and *V. oxycoccos* but their volumes were generally small. The most prevalent vegetable species were *Solanum tuberosum* and *Daucus carota*, whereas *Pyrus communis*, *Malus domestica*, and *Musa* sp. were the most common fruits. Other important plants were *Helianthus annuus* by FO1–2 and volume and *Arachis hypogaea* by volume. The stomachs had higher volumes of plants than the intestines (Mann–Whitney *U*-test, *U* = 1044.000, n = 45, 61, *p* < 0.05). The stomachs had also higher volumes of useful plants (Mann–Whitney *U*-test, *U* = 947.500, n = 44, 60, *p* < 0.05) but their occurrence was lower (*χ*^2^-test, *χ*^*2*^ = 5.583, df = 1, *p* < 0.05).

The identified birds were of 6 species and 6 families, but even the most common avian remains (Phasianidae and Corvidae) were quite rare and found in the gastrointestinal tracts of only 5 animals (see Additional file 6). The stomachs had higher occurrence of birds than the intestines (*χ*^2^-test, *χ*^*2*^ = 6.751, df = 1, *p* < 0.01). The consumed fish were identified as belonging to 3 species and 4 families; the most common family was Percidae followed by Cyprinidae and Esocidae (see Additional file 7). The invertebrates were almost exclusively insects that could not be classified to the species level, and their volumes were very small, higher in the stomachs than in the intestines (Mann–Whitney *U*-test, *U* = 14.500, n = 8, 9, *p* < 0.05; see Additional file 8).

The most voluminous food types in the stomachs were birds (12.3% of the cases), oat (9.9%), raccoon dogs (9.9%), hares (9.9%), and bank voles (9.9%), and in the intestines, oat (13.6%), shrews (13.6%), hares (9.7%), voles/lemmings (9.7%), and raccoon dogs (7.8%). The stomachs of the females had higher Vol1 (Mann–Whitney *U*-test, *U* = 795.500, n = 45, 48, *p* < 0.05) and higher occurrences of raccoon dogs, medium-sized mammals, birds, and cereals (*χ*^2^-test, *χ*^*2*^ = 4.019–8.689, df = 1, *p* < 0.01–0.05) than those of the males, which showed higher RS% of bank voles and total small mammals (Mann–Whitney *U*-test, *U* = 6.000, 137.000, n = 5–26, *p* < 0.05). The diversity index was higher in the stomachs in April compared to Jan–Feb (Kruskal–Wallis ANOVA, *H* = 13.653, df = 5, *p* < 0.05). In early winter, the raccoon dogs consumed more mammals and oat (Kruskal–Wallis ANOVA, *H* = 7.138–14.737, df = 2, *p* < 0.001–0.05) and less fish (stomach: Kruskal–Wallis ANOVA, *H* = 7.977, df = 2, *p* < 0.05). Vol1–2 were the highest in Nov–Dec and decreased in Jan–Feb (Kruskal–Wallis ANOVA, *H* = 15.202–21.445, df = 5, *p* < 0.001–0.01).

The raccoon dogs with fish in their gastrointestinal tracts at the time of sampling had higher proportions of particular n-3 polyunsaturated FA (PUFA; 22:4n-3, 22:5n-3, DHA 22:6n-3), total n-6 PUFA (LA 18:2n-6, 22:4n-6, 22:5n-6), C20–24 saturated FA (SFA), and many C20–22 monounsaturated FA (MUFA) in the sc fat, while the percentages of total MUFA (mainly 18:1n-9) were lower than in the animals without fish (*t*-test, |*t*| = 2.082–4.114; Mann–Whitney *U*-test, *U* = 62.500–119.000; n = 10, 41, *p* < 0.001–0.05). The presence of birds was associated with increased proportions of some C14–17 SFA, a higher n-3/n-6 PUFA ratio, and a decreased 20:1n-9 percentage (*t*-test, |*t*| = 2.153–2.529, n = 14, 15, *p* < 0.05). In PCA, the FAS of the animals, whose gastrointestinal tracts contained fish, were separated from those without fish but there was no clear discrimination according to the other dietary items. The relative proportions of most C12–17 SFA, particular C14–17 MUFA, and C18–20 n-3 PUFA decreased during winter while the percentages of most C18–22 SFA and MUFA together with most C20–22 n-6 PUFA and C21–22 n-3 PUFA increased (*t*-test, |*t*| = 2.071–8.625; Mann–Whitney *U*-test, *U* = 628.500–645.500; n = 39, 46, *p* < 0.001–0.05). The proportions of total MUFA and n-6 PUFA increased and those of total SFA and n-3 PUFA decreased. The preference of FA mobilization can be seen in Additional file 10.

### Home range sizes and habitat preferences

For each animal, 56–208 relocations were obtained during the study periods. The proportion of successful GPS positioning attempts varied from 16 to 62%. The incremental analysis suggested that reliable home range estimates were achieved with approximately 50–140 fixes. Eight area-observation curves approached the asymptote indicating stable home ranges. The average home range size was 5.3 ± 1.1 km^2^ (K95%) and the size of core areas 1.3 ± 0.3 km^2^ (K50%; Table 1). The values were higher when a juvenile male (M5) with a very large home range that expanded across a lake was included in the analyses. Two juveniles performed long dispersions from their initial home ranges to new areas: the sum of distances between successive relocations was 12 km for F2 (11 km as the straight-line distance) and 60 km for M5 (33 km). The year of the study or the sex of the animals did not affect the sizes of home ranges. In contrast, the juveniles had larger home ranges than the adults (K95% with M5: 16.0 ± 8.6 *vs*. 3.5 ± 1.3 km^2^, Mann–Whitney *U*-test, *U* = 5.000, n = 6, *p* < 0.05).

**Table 1:** Sizes of winter home ranges of wild raccoon dogs (n = 12) in eastern Finland

**ID**	**Relocations (n)**	**Kernel 95% (km**^**2**^**)**	**Kernel 50% (km**^**2**^**)**	**Range span (km)**
M1	189	2.0	0.5	2.5
M2	66	10.0	1.6	8.5
M3	188	9.5	2.4	7.1
M4	104	1.0	0.2	3.2
M5	127	58.6	16.9	14.0
M6	208	2.4	0.6	3.3
F1	170	5.6	1.9	4.8
F2^a^	59	10.5	2.3	6.3
F3	103	0.6	0.1	2.4
F4	197	4.5	1.5	5.0
F5	92	9.0	2.4	7.6
F6	56	3.3	1.1	3.6
Mean ± SE^b^	130 ± 18	5.3 ± 1.14	1.3 ± 0.26	4.9 ± 0.65

M = male, F = female, ^a^the mean of two home ranges, ^b^M5 with a very large home range excluded.

**Figure 4:**
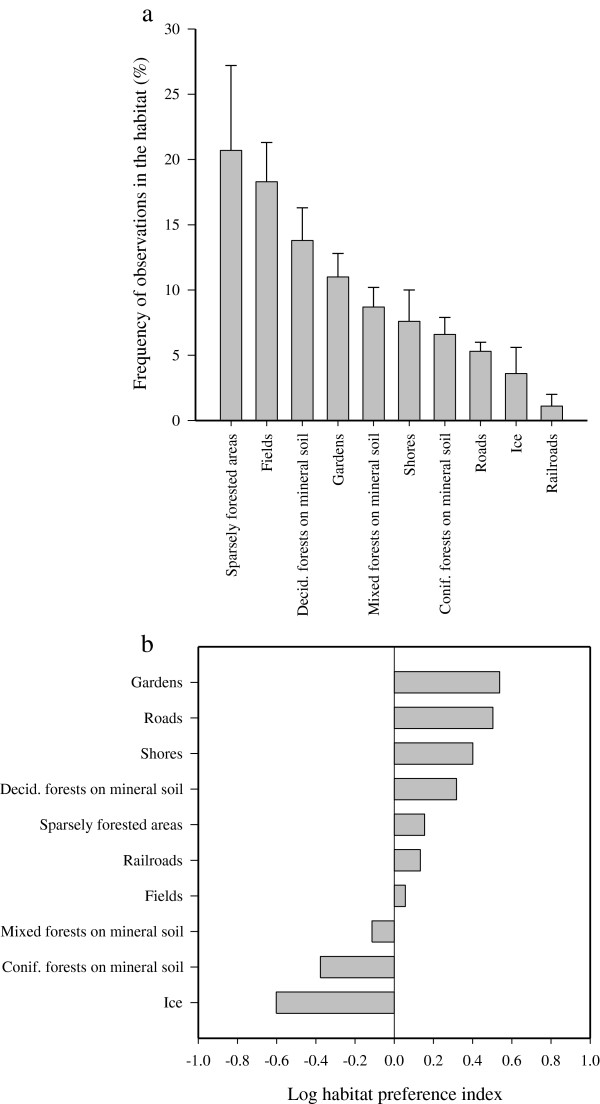
**Wintertime habitat selection and habitat preferences of overwintering raccoon dogs. (a)** The habitat selection (% of observations in the habitat, mean + SE) and **(b)** habitat preferences (0 = equal availability and use, <0 = used less than available, >0 = used more than available; mean values, n = 12).

The proportional availability of habitats was as follows: fields (cereal fields, pastures, fallow farmland; 26% of the main habitats), coniferous forests on mineral soil (17%), mixed forests on mineral soil (13%), sparsely forested areas (canopy cover <30% and/or height <5 m; 13%), ice (lakes, ponds, rivers, ditches; 11%), deciduous forests on mineral soil (7%), shores (lakes, ponds, rivers, ditches; 4%), and gardens (including yards; 4%). The most commonly utilized habitats included sparsely covered areas (20.7 ± 6.5% of the relocations), fields (18.3 ± 3.0%), deciduous forests on mineral soil (13.8 ± 2.5%), gardens (11.0 ± 1.8%), mixed forests on mineral soil (8.7 ± 1.5%), and shores (7.6 ± 2.4%; Figure 4a). When the utilization of biotopes and passages was proportioned to their availability in the area that the animals could have used (1 = equal availability and use, <1 = avoidance, >1 = preference), the following preference was obtained: gardens (3.4 ± 0.7), roads and roadsides (3.2 ± 0.7), shores (2.5 ± 0.7), deciduous forests on mineral soil (2.1 ± 0.5), sparsely forested areas (1.4 ± 0.4), railroads and railroad beds (1.4 ± 1.0), fields (1.1 ± 0.3), mixed forests on mineral soil (0.8 ± 0.2), coniferous forests on mineral soil (0.4 ± 0.1), and ice (0.2 ± 0.1; Figure 4b). The year of the study or the age of the animals did not affect the results, but the females frequented coniferous forests on mineral soil slightly less than the males did (*t*-test, |*t*| = 2.338, n = 6, *p* < 0.05).

### General variables

The average BM was the highest in Dec, reached the nadir in March, and increased between March and April (ANOVA, *F*_*5,78*_ = 17.577, *p* < 0.001). The BMI decreased significantly between Jan and Feb, and the mass of omental fat and the thickness of ventral sc fat decreased from Feb to March (ANOVA, *F*_*5,58–87*_ = 7.953–15.535, *p* < 0.001). The autumnal BMI did not correlate significantly with the number of passive periods of wintering, the total sum of passive days, or the durations of the shortest and longest passive periods. The blood hemoglobin concentration was slightly higher and the mean corpuscular volume lower in spring compared to autumn (Mann–Whitney *U*-test, *U* = 10.500, 9.000, n = 6, 10, *p* < 0.05), but the other parameters in the CBC were not influenced by season (data not shown).

The average wintertime T_a_, calculated for the period with permanent snow cover, was –7.2 ± 0.3°C (minimum –29.0°C, maximum +7.0°C) in 2006–2007, –3.4 ± 0.2°C (–15.0°C, +5.5°C) in 2007–2008, –6.4 ± 0.2°C (–23.0°C, +2.5°C) in 2008–2009, and –11.5 ± 0.3°C (–32.0°C, +5.5°C) in 2009–2010. The maximum snow depths on cultivated land were 45, 70, 41, and 82 cm, respectively.

## Discussion

### Determination of winter sleep periods with change-point analysis

Following the activity patterns of nocturnal and secretive wild mammals is often logistically difficult. GPS tracking is replacing conventional radio tracking and visual observations of, e.g., snow tracks as the principal method for monitoring winter activity, but all these procedures are economically challenging (expensive, labour-intensive, etc.). We developed here a new low-cost, low-effort application of CPA to determine the timing and duration of alternating active and passive periods of overwintering using the raccoon dog as the model species. CPA has been scarcely used in physiological research even though some studies have utilized this method [27,28]. The present experiment is the first to show that CPA can be successfully applied to analyze T_b_ recordings of a passively wintering wild mammal in order to unravel its foraging patterns with potential applications to other northern carnivores.

The positive correlation between the activity scores and T_b_ of the raccoon dogs gives an opportunity to draw conclusions on the timing of passive periods by using the T_b_ data alone. To validate the method, the periods of passivity were determined by both behavioral data and by CPA, and the timing of these periods was compared, i.e., if both methods were able to find the same periods of winter sleep. In individuals that could be tracked for most of the winter, the approximations by CPA and behavioral data shared 91% similarity. As the transitions between activity and passivity are not instant physiological processes, short periods of winter sleep are hard to classify. In the case of more easily defined, longer passive periods of ≥2 days, the similarity was slightly higher (93%). Most of the differences were detected in the transitory days between active and passive wintering. In addition, behavioral data recordings were more sensitive to detect periods of passivity/activity lasting for approximately 1 day.

Although the interrelatedness between the T_b_ and activity scores was clear, they did not correlate perfectly. This may partly result from the method of collecting activity data, as the measurement was not continuous but lasted for 2 min 8 times a day. During data analyses, it was occasionally noted from the GPS fixes that a raccoon dog was away from the den with a relatively high T_b_ but the activity values were close to zero. In these cases, the animal remained fairly immobile for the duration of the activity measurement, although the GPS data indicated that it was not displaying winter sleep at the moment. Due to this, the >90% homology between the two evaluating methods can be considered most satisfactory. Based on the time periods defined by CPA, the T_b_ of the raccoon dogs decreased by 1.0°C from active to passive wintering. The average T_b_ during the seasonal rest (36.5°C) was similar to previous reports on Finnish [5] and Japanese raccoon dogs, *N. p. albus*[6].

In a previous follow-up [5], the duration of (mostly uninterrupted) wintertime passivity determined by radio telemetry was highly variable in eastern Finland (a few days–9 weeks), and the present results enforce the observation that the length of winter sleep can vary prominently depending on the individual. When comparing the data to other species, the average sum of passive days in the present study (53 days) resembles previous estimates on Japanese badgers (*Meles meles anakuma*; [29]) but is less than in Eurasian badgers (*M. meles*) in eastern and northern Europe and in Central Asia [30]. It was also noted in the present study that the raccoon dogs with the highest sums of passive days had also the highest total numbers of passive periods and the longest single passive periods. Previously, it was difficult to distinguish the short passive bouts before and after the principal period of winter sleep as only the latter was clearly visible in the T_b_ curve. CPA seems to be more efficient in pointing out these shorter periods.

Previous studies have indicated that the different types of carnivorean lethargy are highly variable and species-specific. The passivity can be intermittent or continuous, the 24-h rhythm in T_b_ can be maintained or disappear, and the duration and depth of passive bouts can differ significantly. For instance, the T_b_ of bears decreases by 4–5°C, and its circadian rhythmicity is maintained [31]. In Eurasian badgers, the decrease in T_b_ is also pronounced and continuous, but T_b_ does not show a 24-h rhythm [32]. Moreover, the American badger (*Taxidea taxus*; [33]) and the striped skunk (*Mephitis mephitis*; [34]) experience torpor bouts with clear decreases in T_b_. Based on the present and previous studies on raccoon dogs [5,6], the winter rest of the species seems to be the most superficial among these northern carnivores and it is characterized by regular 24-h oscillations of T_b_.

The fluctuations in the T_b_ (see also [5]) and activity scores followed closely the changes in the T_a_. With global warming, winter sleep of the raccoon dog could be interrupted by more frequent warm spells leading to both shorter periods of passivity and decreased total duration of winter rest [2]. The interrelationship of T_b_/activity and T_a_ is observed in several mammals [2,29,30,35-37], and similarly, T_b_ and activity usually increase with day length [29,32,36]. For this reason, the negative covariance between the activity levels and day length in the present study was surprising, and, according to Kauhala et al. [2], day length correlates positively with seasonal activity of raccoon dogs. In the present experiment, the animals were tracked only in winter, and the passive periods occurred mainly in Jan–March when day length was already increasing (Figure 3). This presumably led to the significant negative covariance between these parameters.

Summarizing, T_a_ seems to be an important external factor regulating T_b_ and physical activity levels of the raccoon dog. As T_a_ is liable to the climate change, the duration of winter sleep could be susceptible to global warming with a reduction in the total length of winter sleep and/or a more intermittent pattern of passivity. With the help of CPA, *i*) the timing of passive periods of overwintering can be determined by using T_b_ recordings alone without the burden of VHF and GPS tracking and activity recordings. *ii*) Significantly more individual animals can be recruited by using the simple methods of data loggers and ear tags. *iii*) Hunting can be used as a tool to return ear-tagged individuals with data loggers, allowing *iv*) the extension of follow-up studies to several years with moderate costs. *v*) Applying the T_b_ and CPA methods to other palearctic or nearctic carnivores that are abundant and actively-hunted species would be logistically practical.

### Wintertime diet

As the gastrointestinal tract analysis is limited to the last meals, the FAS of sc fat were also analyzed and compared to the contents of the alimentary canals to obtain data on the more long-term dietary habits [23]. The majority of the animals did consume food in Nov−April, as 84% of the stomachs and 95% of the intestines contained digestible material. The stomach and intestinal compositions were relatively uniform, and the average volume of stomach contents (71 ml) corresponded quite well to previous data (53 ml; [38]). In Jan−Feb, the average food volumes decreased, and the animals relied more on their fat stores. The selective mobilization of FA confirmed our previous results with C12–17 SFA, C14–17 MUFA, and C18–20 n-3 PUFA as the most preferably utilized FA during negative energy balance [23]. Also the diversity index reduced in Jan−Feb as observed previously in different parts of Europe from summer to winter [20,38-42]. Earlier fasting experiments on pigs (*Sus scrofa domestica*) showed that food deprivation of the host decreases the fecundity of roundworms [43] and, thus, the prolonged negative energy balance could have contributed to the observed decrease of intestinal helminths. In the CBC, there were no indications of deleterious health effects caused by wintertime loss of body condition.

As an opportunist, the raccoon dog is very versatile in its selection of food, which in this study consisted of mammals, plants, birds, fish, and invertebrates in this order of importance. Generally, the species can be considered an urban wild animal, as it sometimes lives near or in the habitats humans create. Our raccoon dogs frequently visited farms, yards, gardens, composts, and waste heaps indicated by the occurrence of household waste (e.g., boiled meat and potatoes, imported fruits) and undigestible, man-made material in their gastrointestinal tracts. Also previous studies reported that raccoon dogs utilized compost piles and refuse dumps and that this behavior could be emphasized in winter [20,38,44-47]. In the use of anthropogenic food resources, the raccoon dog resembles several omnivorous canids and mustelids [48]. According to South Korean studies [49], raccoon dogs with insufficient autumnal fattening could leave their principal habitats to search for food in villages increasing the transmission risk of rabies to domestic animals. Not dissimilarly, the raccoon dogs of the present study also utilized human resources. These observations suggest that, if the periods of passivity become shorter in the future [2], the frequency of interactions between raccoon dogs, other wildlife, domestic animals, and humans could increase due to the climate change.

The most common prey items of mammalian origin were small rodents and shrews together with hares, cervids, and raccoon dogs—the latter probably consumed as carrion. The utilization of mammals was the highest in early winter, when it was presumably easier to catch voles and shrews as the snow cover was not yet deep. It is also probable that the vole densities were higher during this period compared to late winter. In earlier studies, voles were important food items for overwintering European raccoon dogs [38,39,41,47,50,51], but the relative proportions of *Myodes* and *Microtus* spp. were highly variable [38,40,41,44]. In the present study, the occurrences were equal in the stomachs but *Microtus* spp. dominated in the intestines, suggesting that *Microtus* could be an even more important genus in the area. Based on the continuous trapping by the Finnish Forest Research Institute during the last 20 years, approximately 60% of the voles have been *M. glareolus* and 40% *M. agrestis* in the study area (Otso Huitu, pers. comm.).

The raccoon dogs, hares, and squirrels were probably consumed as roadkills. The cervids (presumably moose *Alces alces*) had been shot during the hunt and scavenged as discarded offal (rumen, intestines, spleen, kidneys) and hides, as moose are rarely killed by predators or traffic in the study area. Polish and Belarusian raccoon dogs did not rely on medium-sized mammals in winter [40,50], and the importance of hares for Polish, Lithuanian, and Finnish raccoon dogs was previously quite small [38,41,42,51]. The occurrence of raccoon dogs in the diet was not surprising, as similar findings were reported also in an earlier study [38]. Moreover, several papers demonstrated that ungulate carcasses are important as alternative food for European raccoon dogs in winter [20,39-41,47,51,52]. The occurrence of ungulates in the alimentary tracts was not reflected in the FAS, although ruminant fats contain high proportions of SFA and moderate amounts of *trans*-FA due to the microbial biohydrogenation of unsaturated FA in the rumen [53,54]. The raccoon dog fat, however, contained traces of 18:2n*c*9*t*11, which could have derived from moose tissues.

Plants are an important year-round food source for raccoon dogs in all studied geographical regions [47], but there exist scarcely any previous taxonomical data on the diversity of plant-based food items. In the present study, this was realized by the detailed analysis of seeds in the gastrointestinal contents. In agricultural landscapes of Germany, maize (*Zea mays*) was the most popular winter food item [47], whereas in Finland oat was one of the most common species, especially in early winter. Its presence indicated that the animals visited fields, arable lands, grain storages, and/or feeding stations of game animals. This was supported by the observations of common weeds, such as *Chenopodium album*. The occurrence of cereal grains in the winter food has been established also previously [20,38,44].

Bird feeders—a food source used by urban foxes [55]—were also visited by raccoon dogs as, in addition to oat often present in bird feeds, their gastrointestinal tracts contained seeds of *H. annuus*, *A. hypogaea*, *Sorghum* sp., *Panicum miliaceum, Setaria italica*, and *Linum usitatissimum*, plant species not cultivated in the study area. Berries rich in carbohydrates were important food items for raccoon dogs during the autumnal fat gain [38,56]. Also the winter diet contained berries, especially *S. aucuparia*, *V. vitis-idaea*, and *V. oxycoccos* (see also [40,42]), all of which are well-preserved through winter. Our study animals also consumed dropped or discarded apples together with pears and bananas presumably originating from compost piles.

The occurrence of birds in the winter diet was relatively similar or higher in the present experiment compared to previous European studies [38,40-42,44,47,50,51]. The consumed specimens were mainly gallinaceous birds, waterfowl, and corvids [38,44], and most of the identified species were year-round residents. In addition to *Anas* sp., only two of them (*Tetrastes bonasia*, *Lyrurus tetrix*) could be considered game bird species and, thus, the results suggest that in the study area the raccoon dog, albeit an introduced species, cannot be considered a significant threat to wildfowl in winter (see also [38,44]).

Ice-fishing is popular in the study area, especially in late winter−early spring, when the occurrence of fish in the diet increased and raccoon dog tracks were observed on ice [5,38]. Most specimens that could be identified belonged to Percidae and Cyprinidae ([44] for the snow-free period). Presumably this does not represent any preference of the raccoon dogs but reflects the species that are the most likely to be discarded by fishers. Previous European studies reported low fish consumption by overwintering raccoon dogs [38,40,42,47] but, in some areas, the importance of fish increased in early spring [20,38]. According to PCA, the occurrence of fish separated the FAS of the animals. It is impossible to know, if the individuals with remains of fish in their alimentary tracts foraged regularly on fish left by fishermen or households, but the FAS analysis suggests that this could be the case. Freshwater fish can contain high amounts of, e.g., n-3 and n-6 PUFA [57], which could explain the higher proportions of LA and longer-chain PUFA in the fat tissue of these individuals. The DHA/LA ratio, correlating with aquatic food web exploitation [58], averaged 0.085 in the raccoon dogs—slightly higher than calculated for terrestrial mustelids and felids but approximately at the same level as in semi-aquatic mustelids [58,59]. This suggests that also the raccoon dog foraged on freshwater food during winter, even though its DHA/LA ratio was below that of the European otter (*Lutra lutra*).

Invertebrates were of minor importance for the overwintering raccoon dogs, and amphibians or reptiles were not detected in any of the samples. In previous investigations, the occurrences of these food items were similar or higher than in the present study [38,40-42,44,47,51]. As the winters can be harsh in eastern Finland, it is difficult for the animals to find, e.g., earthworms or anurans under deep snow, ice, and ground frost. In the more temperate climate of Japan, insects are an important food class year-round [45]. It is possible that some insects (and some other dietary items) found in the gastrointestinal tracts in the present study could have been predigested by the prey animals.

To sum up, *i*) overwintering raccoon dogs are opportunists and active participants in the food web. *ii*) Mammals (arvicolines, shrews, cadavers) and plants (oat, berries) together with birds and discarded fish comprise the most important winter food items. *iii*) Raccoon dogs utilize anthropogenic food resources (cereals, garden supply, household waste, spilled bird seeds, carcasses of hunted moose) similar to several other carnivores, and *iv*) the occurrence of fish in the diet can be detected in the FAS of overwintering raccoon dogs.

### Size of winter home ranges and habitat preferences

There is a limited amount of data on comparisons of home range sizes estimated by VHF radio telemetry and GPS tracking. In the present study, the sizes of winter home ranges determined by GPS averaged 5.3 km^2^ (K95%) and the core areas 1.3 km^2^. These estimates were similar or higher than previous measurements conducted with VHF radio telemetry in Finland during the snow-free season (K95%: 1.0–3.9 km^2^; K50/60%: 0.2–0.7 km^2^; [5,60-63]) and in winter (K95%: 3.7 km^2^; K50%: 0.5 km^2^; [5]). In a New Zealand study, Recio et al*.*[64] determined the home range size of feral cats (*Felis catus*) with GPS and compared it to results obtained by radio telemetry. They concluded that the home range sizes were of a similar magnitude or higher when using GPS. Furthermore, Medri & Mourão [65] reported for the giant anteater (*Myrmecophaga tridactyla*) that a home range recorded with GPS over 9 days could be larger than a range obtained with VHF tracking over 252 days. These data support our results showing similar or larger winter home range sizes than estimated previously for raccoon dogs in the same study area with traditional tracking methods [5].

In Germany, dispersal of juveniles was rare in winter [66], but our results suggest that they could be more mobile and have larger home ranges than adults during the cold season. The size of the stable postdispersal home range of M5 (K95%: 59 km^2^) seemed exceptionally large but, in fact, an even higher value was reported for a juvenile German raccoon dog (K100%: >150 km^2^), which roamed solitarily in search of a suitable habitat and a mate [66]. Supported by data of Åhlén and Dahl [67], GPS can have better potential than radio telemetry to unravel rapid long-distance dispersions of raccoon dogs, as these can occur unexpectedly towards an unpredictable direction. They can cause the loss of animals from conventional VHF telemetry especially in rugged terrain, where the distance for receiving signals can be fairly short, while dispersion presents no obstacle for GPS.

The study area was sparsely-populated small-scale mosaic with little patches of fields, forests, and gardens. This can have methodological significance, as even small inaccuracies in GPS fixes could switch the position of the relocation to the adjacent habitat. Also, when the density of forest canopy increases, the GPS fix attempts may be less successful [1], which could cause a bias in habitat selection data. The observation success rates varied greatly between individuals (16–62%), and this could have been partly derived from the differences in the proportion of forest habitats in the home ranges, although the activity levels presumably affected the observation success rates the most. Moreover, the significance of the canopy is presumably lower in winter with no foliage in the deciduous trees. It is not possible to state with certainty why the % of successful GPS fixes was lower than in a previous follow-up of raccoon dogs in Greater Tokyo in Dec and March—98% in a relatively open area and 70% in a mosaic area [68]. Potential explanations include differences in climates (lack of permanent snow cover in the Tokyo region), in underground hiding behavior of raccoon dogs combined with the characteristics of the habitats, and in coverage of GPS.

The raccoon dog is an ecological generalist. The biotopes preferred by the overwintering individuals were gardens, shores, deciduous forests, and sparsely forested areas. These findings fit relatively well with the results of the dietary analyses discussed above. The wintertime habitat choice of the species has been scarcely studied, and the comparisons between experiments can be complicated due to the different classification of habitat types and varying habitat availabilities in other countries. For instance, German raccoon dogs showed almost neutral preference for forests, small woods, maize fields, hedges, reeds, and meadows but avoided open farmland, water surfaces, and human settlement [69].

Previously, raccoon dogs were observed to prefer gardens and yards during the snow-free season in Finland [60,63], and they seemed to favor man-made biotopes of this type also in winter. This finding is supported by the gastrointestinal tract contents with fruits (apples), berries (rowanberries, chokeberries), bird feeds, and household waste from compost piles (potatoes, carrots, and their peals, banana skins, pear seeds, etc.). Anthropogenic food resources provide many carnivores an abundant, relatively stable, and highly concentrated food source and, in some cases, larger home ranges in rural areas may be associated with lower availability of these food items compared to urban and suburban sites [70]. Raccoon dogs favored also shores during the snow-free season in Finland [56,62]. Shores were presumably preferred as they provide food (e.g., frogs), shelter, and quick escape into water when attacked. In winter, the popularity of frozen lake and pond shores may be related to the availability of discarded fish as discussed above. The available area of lake ice was large in the potential home range of some individuals, but this habitat was not among the most preferred, even though >20% of the relocations of M5 were on ice.

When discussing forest types, raccoon dogs were previously documented to favor deciduous and mixed forests and to avoid coniferous forests in the snow-free season in Finland [60-63]. In Lithuania, overwintering raccoon dogs selected spruce/mixed coniferous forests over deciduous/mixed forests, while pine forests with poor food resources were avoided [42,71]. In the present study, deciduous forests were clearly preferred over mixed or coniferous forests. The unpopularity of low-open coniferous woodland may result from the lack of protective undergrowth, although these sites could provide animals with, e.g., berries. One possibility could also be the difficulty to obtain a GPS fix through the coniferous, often snow-covered canopy in winter. The GPS collared raccoon dogs of the present experiment utilized wetland habitats of the study area hardly at all, unlike in Lithuania and Belarus [40,42,71], but cranberries can be available also on pond and lake shores covered by a zone of *Sphagnum* moss. The cranberries found in the gastrointestinal tracts presumably originated from these sites and from household waste.

One reason for the preference of sparsely forested areas could be the fact that the most frequently used winter den of a raccoon dog pair was situated in this habitat type and, as a result, a major portion of their GPS fixes was located close to the den. Sparsely forested sites were structurally complex as they consisted of, e.g., sapling stands and clear-cuts with abundant undergrowth providing the animals with shelter and small prey. Also lingonberries could be available in clear-cut areas. Furthermore, raccoon dogs commonly rest under lower branches of young spruces *Picea abies*, which could be one explanation to the popularity of sparsely forested areas. In contrast to the present results, sapling stands and clear-cuts were not preferred during the snow-free season in Finland [60]. Even though almost 20% of the relocations of the present study were in fields, their high areal proportion in the study district led to almost neutral preference, which is similar to observations on the utilization of maize fields in Germany [69]. Fields were among the preferred habitats by Finnish raccoon dogs in the snow-free season [60,62,63], and they were probably good sources of cereals and small mammalian prey [16] for overwintering raccoon dogs, as well.

Many mammalian species are disturbed by human infrastructure such as roads. They have negative ecological effects by forming movement barriers and by causing habitat fragmentation and loss, disturbance, pollution, and mortality [72]. Roadsides can also provide refuges, new habitats, and movement corridors, although many carnivores tend to avoid the presence of roads [73,74]. Raccoon dogs have high mortality due to traffic [66], but the use of groomed roads and railroad beds indicates their possible benefits as travel routes due to thinner snow with a more supportive surface. This could decrease the locomotory costs, as raccoon dogs have small footpads and they sink easily into soft, deep snow. Also other mammals use railway tracks to facilitate traveling during periods of deep snow cover [75].

The emergence of rodents from hibernation became significantly earlier in the Colorado Rocky Mountains from 1976 to 1999 [76]. Similar long-term follow-ups would be useful also in other passively wintering species, such as the raccoon dog, which is likely to benefit from the extending growing season and shorter winters. According to climate change scenarios [77], T_a_ in Finland will increase during all seasons but especially in winter. Towards the end of the present century, the number of frost days will decrease by 40–80 days, the period of snow cover will shorten by >1 to 2 months, and snow depth in midwinter will decrease to about 33–70% of that at present. Passivity of the raccoon dog will probably become shorter and more intermittent during milder winters [2]. Abundance of the raccoon dog and other medium-sized predators can increase and their distribution expand to north [77]. This could also influence the occurrence and distribution of zoonotic diseases and parasites they transmit.

Summarizing, *i*) the wintertime home ranges determined by GPS could give a more accurate estimation of the home range sizes of raccoon dogs compared to traditional methods. *ii*) The preferred habitat types were gardens, shores, deciduous forests, and sparsely forested areas, while fields had close to neutral preference. These data support the results of the dietary analyses. *iii*) The raccoon dogs used roads and railroads as wintertime travel routes.

## Conclusions

A multi-faceted approach was used to investigate the wintertime ecophysiology of the raccoon dog—a suitable and abundant model for winter sleep studies. By utilizing GPS tracking, activity sensors, T_b_ recordings, CPA, home range, habitat, and dietary analyses, the impact of this alien species on wintertime food webs was assessed. The timing of passive bouts was initially determined with multiple methods and compared to T_b_ recordings after analyzing them with CPA. To sum up, *i*) raccoon dogs displayed significant wintertime mobility, and the home range sizes determined by GPS were similar or larger than those acquired previously by radio tracking. The preferred habitats were gardens, shores, deciduous forests, and sparsely forested areas, while fields had close to neutral preference. Roads and railroads were utilized as travel routes. *ii*) Overwintering raccoon dogs were active participants in the food web, and their opportunistic feeding depended partly on human activity. Mammals (arvicolines, shrews, carcasses), plants (oat, berries), birds, and discarded fish were the most important dietary items together with anthropogenic foods (compost waste, bird feeds, agricultural products). The consumption of fish could be detected in FAS. *iii*) T_a_ that is liable to the climate change appeared to be an important external factor influencing T_b_ during overwintering, while the interactions of T_b_ with snow depth and day length were nonsignificant. The physical activity levels had positive covariance with T_a_ and negative covariance with the depth of snow. Winter sleep of the raccoon dog could be susceptible to global warming with a reduction in the total length of winter sleep and/or a more intermittent pattern of passivity. *iv*) CPA based on repeated T_b_ measurements offers a reliable and economical method to investigate the bouts of passivity with potentially significant applications in, e.g., climate change research.

## Authors’ contributions

AMM, PNieminen, JAsikainen, and PNiemelä acquired funding, designed, and coordinated the study. MA developed the change-point analysis application. KI, TL, PH, VV, JE, JI, MK, JAho, AMM, PNieminen, and JAsikainen carried out the dietary and biochemical analyses. AMM, PNieminen, and JAsikainen conducted the GPS tracking, home range, and habitat analyses. PNieminen performed the statistical analyses. AMM drafted the manuscript with the help of PNieminen. All authors read and approved the final manuscript.

## Supplementary Material

Additional file 1 Monthly total volumes of all digestible food items in the stomachs and intestines of wild raccoon dogs. Click here for file

Additional file 2 Diversity of mammals in the stomachs of wild raccoon dogs. Click here for file

Additional file 3 Diversity of mammals in the intestines of wild raccoon dogs. Click here for file

Additional file 4 Diversity of plants in the stomachs of wild raccoon dogs. Click here for file

Additional file 5 Diversity of plants in the intestines of wild raccoon dogs. Click here for file

Additional file 6 Diversity of birds in the stomachs and intestines of wild raccoon dogs. Click here for file

Additional file 7 Diversity of fish in the stomachs and intestines of wild raccoon dogs. Click here for file

Additional file 8 Diversity of invertebrates in the stomachs and intestines of wild raccoon dogs. Click here for file

Additional file 9 Distribution of dietary items in the stomachs and intestines of wild raccoon dogs. Click here for file

Additional file 10 Relative changes in the proportions of selected adipose tissue fatty acids during winter in wild raccoon dogs. Click here for file

## References

[B1] Rodgers AR Sibbald AM Tracking animals with GPS: the first 10 years. Tracking Animals with GPS. An International Conference held at the Macaulay Land Use Research Institute: 12–13 March 2001 2001 Gordon IJ: Aberdeen 1 10

[B2] Kauhala K Holmala K Schregel J Seasonal activity patterns and movements of the raccoon dog, a vector of diseases and parasites, in southern Finland. Mamm Biol 2007 72 342 353

[B3] Mikkola M Management Plan of the Raccoon Dog 2011 Helsinki: Suomen riistakeskus In Finnish

[B4] Melis C Herfindal I Kauhala K Andersen R Høgda K-A Predicting animal performance through climatic and plant phenology variables: the case of an omnivore hibernating species in Finland. Mamm Biol 2010 75 151 159

[B5] Mustonen A-M Asikainen J Kauhala K Paakkonen T Nieminen P Seasonal rhythms of body temperature in the free-ranging raccoon dog (*Nyctereutes procyonoides*) with special emphasis on winter sleep Chronobiol Int 2007 24 1095 1107 10.1080/07420520701797999 18075801

[B6] Kitao N Fukui D Hashimoto M Osborne PG Overwintering strategy of wild free-ranging and enclosure-housed Japanese raccoon dogs (*Nyctereutes procyonoides albus*) Int J Biometeorol 2009 53 159 165 10.1007/s00484-008-0199-7 19101736

[B7] van Marken Lichtenbelt WD Daanen HAM Wouters L Fronczek R Raymann RJEM Severens NMW Van Someren EJW Evaluation of wireless determination of skin temperature using iButtons Physiol Behav 2006 88 489 497 10.1016/j.physbeh.2006.04.026 16797616

[B8] Nieminen P Saarela S Pyykönen T Asikainen J Mononen J Mustonen A-M Endocrine response to fasting in the overwintering captive raccoon dog (*Nyctereutes procyonoides*) J Exp Zool 2004 301A 919 929 10.1002/jez.a.126 15562452

[B9] Kauhala K Helle E Age determination of the raccoon dog in Finland Acta Theriol 1990 35 321 329

[B10] McNay RS Morgan JA Bunnell FL Characterizing independence of observations in movements of Columbian black-tailed deer J Wildl Manage 1994 58 422 429 10.2307/3809312

[B11] Rooney SM Wolfe A Hayden TJ Autocorrelated data in telemetry studies: time to independence and the problem of behavioural effects Mammal Rev 1998 28 89 98 10.1046/j.1365-2907.1998.00028.x

[B12] de Solla SR Bonduriansky R Brooks RJ Eliminating autocorrelation reduces biological relevance of home range estimates J Anim Ecol 1999 68 221 234 10.1046/j.1365-2656.1999.00279.x

[B13] Worton BJ Kernel methods for estimating the utilization distribution in home-range studies Ecology 1989 70 164 168 10.2307/1938423

[B14] Kenward RE South AB Walls SS Ranges6 v1.2: For the Analysis of Tracking and Location Data 2003 Wareham: Anatrack Ltd

[B15] Kauhala K Helle E Taskinen K Home range of the raccoon dog (*Nyctereutes procyonoides*) in southern Finland J Zool 1993 231 95 106 10.1111/j.1469-7998.1993.tb05355.x

[B16] Siivonen L Sulkava S Pohjolan nisäkkäät (Mammals of Northern Europe) 1994 Helsinki: Otava In Finnish

[B17] Cappers RTJ Bekker RM Jans JEA Digitale Zadenatlas van Nederland 2006 Groningen: Barkhuis Publishing & Groningen University Library

[B18] Kottelat M Freyhof J Handbook of European Freshwater Fishes 2007 Cornol: Publications Kottelat

[B19] Oates DW Krings LM Ditz KL Field manual for the identification of selected North American freshwater fish by fillets and scales. Nebraska Technical Series No. 19 1993 Lincoln: Nebraska Game and Parks Commission Available online at [http://icwdm.org/inspection/Fish/FishManual.pdf]. Accessed on March 25 2012

[B20] Sidorovich VE Solovej IA Sidorovich AA Dyman AA Seasonal and annual variation in the diet of the raccoon dog *Nyctereutes procyonoides* in northern Belarus: the role of habitat type and family group Acta Theriol 2008 53 27 38 10.1007/BF03194276

[B21] Day MG Identification of hair and feather remains in the gut and faeces of stoats and weasels J Zool 1966 148 201 217

[B22] Deedrick DW Koch SL Microscopy of hair Part II: a practical guide and manual for animal hairs. Forensic Science Communications 2004 6 available online at [http://www.fbi.gov/about-us/lab/forensic-science-communications/fsc/july2004/research/2004_03_research02.htm]. Accessed on Jan 19 2012

[B23] Mustonen A-M Asikainen J Aho J Nieminen P Selective seasonal fatty acid accumulation and mobilization in the wild raccoon dog (*Nyctereutes procyonoides*) Lipids 2007 42 1155 1167 10.1007/s11745-007-3118-5 17926077

[B24] Taylor WA Change-point analysis: A powerful new tool for detecting changes[http://www.variation.com/cpa/tech/changepoint.html]. Accessed on Feb 3 2012

[B25] Taylor WA A pattern test for distinguishing between autoregressive and mean-shift data[http://www.variation.com/cpa/tech/pattern.html]. Accessed on Feb 3 2012

[B26] Kvalheim OM Karstang TV A general-purpose program for multivariate data analysis Chemometr Intell Lab 1987 2 235 237 10.1016/0169-7439(87)80101-6

[B27] Ackerman JT Takekawa JY Kruse KL Orthmeyer DL Yee JL Ely CR Ward DH Bollinger KS Mulcahy DM Using radiotelemetry to monitor cardiac response of free-living tule greater white-fronted geese (*Anser albifrons elgasi*) to human disturbance Wilson Bulletin 2004 116 146 151 10.1676/03-110

[B28] Cassidy M Mazzone P Oliviero A Insola A Tonali P Di Lazzaro V Brown P Movement-related changes in synchronization in the human basal ganglia Brain 2002 125 1235 1246 10.1093/brain/awf135 12023312

[B29] Tanaka H Winter hibernation and body temperature fluctuation in the Japanese badger, *Meles meles* anakuma Zool Sci 2006 23 991 997 10.2108/zsj.23.991 17189911

[B30] Kowalczyk R Jędrzejewska B Zalewski A Annual and circadian activity patterns of badgers (*Meles meles*) in Białowieża primeval forest (eastern Poland) compared with other Palaearctic populations J Biogeography 2003 30 463 472 10.1046/j.1365-2699.2003.00804.x

[B31] Hissa R Siekkinen J Hohtola E Saarela S Hakala A Pudas J Seasonal patterns in the physiology of the European brown bear (*Ursus arctos arctos*) in Finland Comp Biochem Physiol 1994 109A 781 791 10.1016/0300-9629(94)90222-4 8529017

[B32] Fowler PA Racey PA Overwintering strategies of the badger, *Meles meles*, at 57 °N J Zool 1988 214 635 651 10.1111/j.1469-7998.1988.tb03763.x

[B33] Harlow HJ Torpor and other physiological adaptations of the badger (*Taxidea taxus*) to cold environments Physiol Zool 1981 54 267 275

[B34] Hwang YT Larivière S Messier F Energetic consequences and ecological significance of heterothermy and social thermoregulation in striped skunks (*Mephitis mephitis*) Physiol Biochem Zool 2007 80 138 145 10.1086/509211 17160886

[B35] Aleksiuk M Stewart AP Food intake, weight changes and activity of confined striped skunks (*Mephitis mephitis*) in winter Am Midland Nat 1977 98 331 342 10.2307/2424984

[B36] Bevanger K Brøseth H Body temperature changes in wild-living badgers Meles meles through the winter Wildlife Biol 1998 4 97 101

[B37] Kanda LL Fuller TK Friedland KD Temperature sensor evaluation of opossum winter activity Wildl Soc Bull 2005 33 1425 1431 10.2193/0091-7648(2005)33[1425:TSEOOW]2.0.CO;2

[B38] Kauhala K Kaunisto M Helle E Diet of the raccoon dog, *Nyctereutes procyonoides*, in Finland Z Säugetierkunde 1993 58 129 136

[B39] Kobylińska J The red fox and raccoon dog in wetlands of the Biebrza river valley — food composition and burrow use. J Wildl Res 1996 1 186 189

[B40] Sidorovich VE Polozov AG Lauzhel GO Krasko DA Dietary overlap among generalist carnivores in relation to the impact of the introduced raccoon dog *Nyctereutes procyonoides* on native predators in northern Belarus Z Säugetierkunde 2000 65 271 285

[B41] Baltrūnaitė L Diet composition of the red fox (*Vulpes vulpes* L.), pine marten (*Martes martes* L.) and raccoon dog (*Nyctereutes procyonoides* Gray) in clay plain landscape, Lithuania Acta Zool Lituanica 2002 12 362 368 10.1080/13921657.2002.10512525

[B42] Baltrūnaitė L Diet and winter habitat use of the red fox, pine marten and raccoon dog in Dzūkija National Park, Lithuania Acta Zool Lituanica 2006 16 46 53 10.1080/13921657.2006.10512709

[B43] Petkevičius S Nansen P Stephenson L The effect of fasting on *Ascaris suum* and *Oesophagostomum spp*. In growing pigs Int J Parasitol 1997 27 431 437 10.1016/S0020-7519(96)00190-7 9184936

[B44] Viro P Mikkola H Food composition of the raccoon dog Nyctereutes procyonoides Gray, 1834 in Finland Z Säugetierkunde 1981 46 20 26

[B45] Sasaki H Kawabata M Food habits of the raccoon dog *Nyctereutes procyonoides viverrinus* in a mountainous area of Japan J Mamm Soc Japan 1994 19 1 8

[B46] Kauhala K Laukkanen P von Rége I Summer food composition and food niche overlap of the raccoon dog, red fox and badger in Finland Ecography 1998 21 457 463 10.1111/j.1600-0587.1998.tb00436.x

[B47] Sutor A Kauhala K Ansorge H Diet of the raccoon dog *Nyctereutes procyonoides* – a canid with an opportunistic foraging strategy Acta Theriol 2010 55 165 176 10.4098/j.at.0001-7051.035.2009

[B48] Fedriani JM Fuller TK Sauvajot RM Does availability of anthropogenic food enhance densities of omnivorous mammals? An example with coyotes in southern California. Ecography 2001 24 325 331

[B49] Kim C-H Lee C-G Yoon H-C Nam H-M Park C-K Lee J-C Kang M-I Wee S-H Rabies, an emerging disease in Korea J Vet Med B 2006 53 111 115 10.1111/j.1439-0450.2006.00928.x 16629721

[B50] Reig S Jędrzejewski W Winter and early spring food of some carnivores in the Białowieża National Park, eastern Poland Acta Theriol 1988 33 57 65

[B51] Jędrzejewski W Jędrzejewska B Szymura A Food niche overlaps in a winter community of predators in the Białowieża primeval forest, Poland Acta Theriol 1989 34 487 496

[B52] Selva N Jedrzejewska B Jedrzejewski W Wajrak A Scavenging on European bison carcasses in Bialowieza primeval forest (eastern Poland) Écoscience 2003 10 303 311

[B53] Tanhuanpää E Pulliainen E Major fatty acid composition of some organ fats in the moose (Alces alces) in northeastern Lapland Ann Zool Fennici 1975 12 148 155

[B54] Aro A Antoine JM Pizzoferrato L Reykdal O van Poppel G *Trans* fatty acids in dairy and meat products from 14 European countries: the TRANSFAIR study J Food Compos Anal 1998 11 150 160 10.1006/jfca.1998.0570

[B55] Contesse P Hegglin D Gloor S Bontadina F Deplazes P The diet of urban foxes (*Vulpes vulpes*) and the availability of anthropogenic food in the city of Zurich, Switzerland Mamm Biol 2004 69 81 95

[B56] Kauhala K Habitat use of raccoon dogs, *Nyctereutes procyonoides*, in southern Finland Z Säugetierkunde 1996 61 269 275

[B57] Steffens W Effects of variation in essential fatty acids in fish feeds on nutritive value of freshwater fish for humans Aquaculture 1997 151 97 119 10.1016/S0044-8486(96)01493-7

[B58] Koussoroplis A-M Lemarchand C Bec A Desvilettes C Amblard C Fournier C Berny P Bourdier G From aquatic to terrestrial food webs: decrease of the docosahexaenoic acid/linoleic acid ratio Lipids 2008 43 461 466 10.1007/s11745-008-3166-5 18335265

[B59] Zalewski K Martysiak-Żurowska D Iwaniuk M Nitkiewicz B Stołyhwo A Characterization of fatty acid composition in Eurasian badger (*Meles meles*) Polish J Environ Stud 2007 16 645 650

[B60] Kauhala K Holmala K Optimal radio-tracking strategy – the best results with the least effort? Acta Theriol 2008 53 333 341 10.1007/BF03195194

[B61] Holmala K The community of medium-sized carnivores: the interactions between species, habitats and rabies. PhD thesis 2009 University of Helsinki, Department of Biological and Environmental Sciences

[B62] Holmala K Kauhala K Habitat use of medium-sized carnivores in southeast Finland — key habitats for rabies spread? Ann Zool Fennici 2009 46 233 246

[B63] Kauhala K Auttila M Habitat preferences of the native badger and the invasive raccoon dog in southern Finland Acta Theriol 2010 55 231 240 10.4098/j.at.0001-7051.040.2009

[B64] Recio MR Mathieu R Maloney R Seddon PJ First results of feral cats (*Felis catus*) monitored with GPS collars in New Zealand New Zeal J Ecol 2010 34 288 296

[B65] Medri IM Mourão G Home range of giant anteaters (*Myrmecophaga tridactyla*) in the Pantanal wetland, Brazil J Zool 2005 266 365 375 10.1017/S0952836905007004

[B66] Drygala F Zoller H Stier N Roth M Dispersal of the raccoon dog *Nyctereutes procyonoides* into a newly invaded area in Central Europe Wildl Biol 2010 16 150 161 10.2981/08-076

[B67] Åhlén P-A Dahl F Mårdhundar ute på långvandring Svensk Jakt 2011 3 issue 16 In Swedish

[B68] Takeuchi T Matsuki R Nashimoto M GPS cell phone tracking in the Greater Tokyo Area: a field test on raccoon dogs. Urban Ecosyst 2012 15 181 193 10.1007/s11252-011-0200-9

[B69] Drygala F Stier N Zoller H Boegelsack K Mix HM Roth M Habitat use of the raccon dog (*Nyctereutes procyonoides*) in north-eastern Germany Mamm Biol 2008 73 371 378

[B70] Prange S Gehrt SD Wiggers EP Influences of anthropogenic resources on raccoon (*Procyon lotor*) movements and spatial distribution J Mammal 2004 85 483 490 10.1644/BOS-121

[B71] Baltrūnaitė L Winter habitat use, niche breadth and overlap between the red fox, pine marten and raccoon dog in different landscapes of Lithuania Folia Zool 2010 59 278 284

[B72] Seiler A Ecological Effects of Roads, a Review. Introductory Research Essay no 9 2001 Uppsala: Swedish University of Agricultural Sciences, Department of Conservation Biology

[B73] McLellan BN Shackleton DM Grizzly bears and resource-extraction industries: effects of roads on behaviour, habitat use and demography J Appl Ecol 1988 25 451 460 10.2307/2403836

[B74] Van Dyke FG Brocke RH Shaw HG Ackerman BB Hemker TP Lindzey FG Reactions of mountain lions to logging and human activity J Wildl Manage 1986 50 95 102 10.2307/3801496

[B75] Klein DR Reaction of reindeer to obstructions and disturbances Science 1971 173 393 398 10.1126/science.173.3995.393 17770437

[B76] Inouye DW Barr B Armitage KB Inouye BD Climate change is affecting altitudinal migrants and hibernating species Proc Natl Acad Sci 2000 97 1630 1633 10.1073/pnas.97.4.1630 10677510 PMC26486

[B77] Marttila V Granholm H Laanikari J Yrjölä T Aalto A Heikinheimo P Honkatukia J Järvinen H Liski J Merivirta R Paunio M Finland’s National Strategy for Adaptation to Climate Change 2005 Helsinki: Ministry of Agriculture and Forestry

