# Supplementary material for: Application of change-point analysis to determine winter sleep patterns of the raccoon dog (Nyctereutes procyonoides) from body temperature recordings and a multi-faceted dietary and behavioral study of wintering
Source: BMC Ecol. 2012 Dec 13;12:27. doi: 10.1186/1472-6785-12-27 (PMC3549453; doi:10.1186/1472-6785-12-27)

Additional file 1. Monthly total volumes of all digestible food items (baits excluded) in the stomachs and intestines. Mean + SE, n = 20 (Nov), 20 (Dec), 24 (Jan), 6 (Feb), 19 (March) and 4 (April). Means with dissimilar letters are statistically different from each other within the stomachs or the intestines (Kruskal–Wallis ANOVA, $p<0.01$).

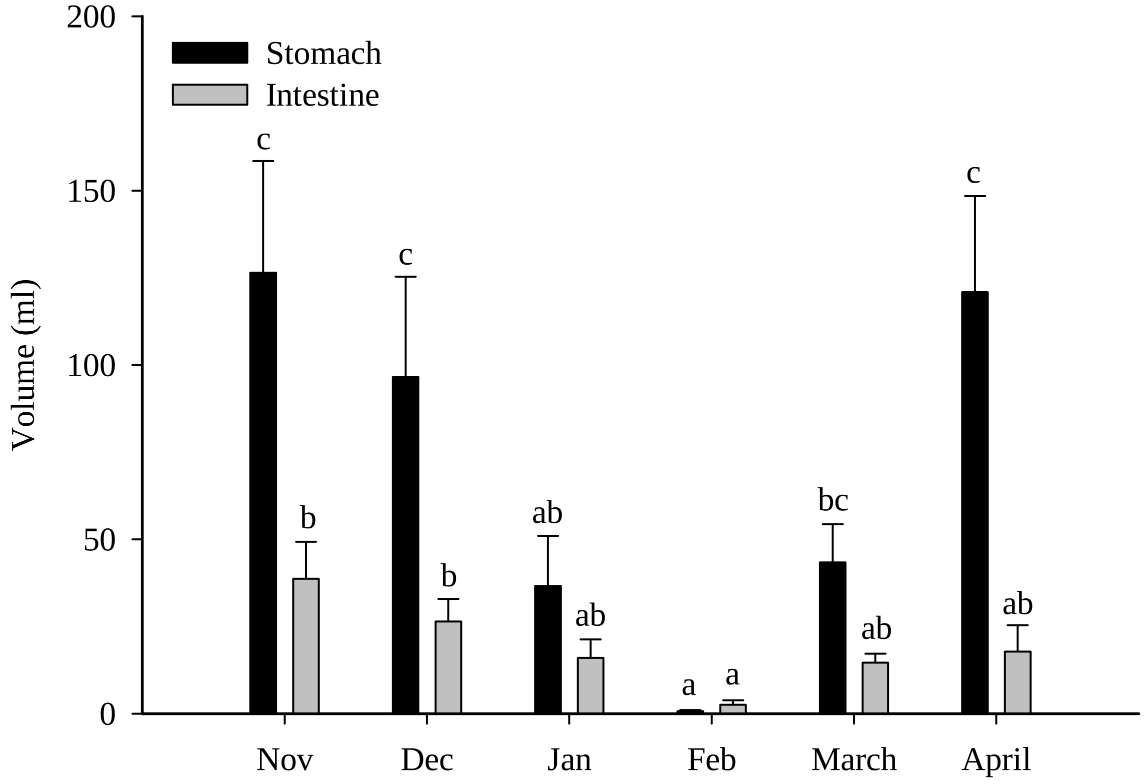

Supplement: Additional file 1 — Monthly total volumes of all digestible food items in the stomachs and intestines of wild raccoon dogs. [file 1472-6785-12-27-S1.pdf]
