# Supplementary material for: Application of change-point analysis to determine winter sleep patterns of the raccoon dog (Nyctereutes procyonoides) from body temperature recordings and a multi-faceted dietary and behavioral study of wintering
Source: BMC Ecol. 2012 Dec 13;12:27. doi: 10.1186/1472-6785-12-27 (PMC3549453; doi:10.1186/1472-6785-12-27)
Supplement: Additional file 2 — Diversity of mammals in the stomachs of wild raccoon dogs. [file 1472-6785-12-27-S2.pdf]

Additional file 2. Diversity of mammals in the stomachs of wild raccoon dogs.

		N	FO1 (%)	FO2 (%)	Volume (ml)	RS (%)
∑ Small mammals		43	46.2	16.3	17.3 ± 5.3	48.8 ± 6.4
	Sorex araneus	2	2.2	0.8	7.7 ± 2.7	52.1 ± 47.9
	Sorex spp.	8	8.6	3.0	4.8 ± 2.5	30.4 ± 15.4
	Talpa europaea	1	1.1	0.4	30.0	24.9
	Myodes glareolus	14	15.1	5.3	10.1 ± 4.1	56.8 ± 10.3
	Microtus agrestis	8	8.6	3.0	17.0 ± 10.1	46.8 ± 13.6
	Microtus spp.	7	7.5	2.7	6.1 ± 1.8	42.7 ± 17.5
	Myopus schisticolor	1	1.1	0.4	13.5	55.3
	Unidentified Arvicolinae	2	2.2	0.8	3.5 ± 2.3	12.2 ± 9.9
	Unidentified Muroidea	1	1.1	0.4	0.2	0.1
	Unidentified Murinae	1	1.1	0.4	10.4	42.6
	Rattus norvegicus	1	1.1	0.4	175.0	96.7
	Sciurus vulgaris	5	5.4	1.9	26.8 ± 16.0	7.7 ± 5.0
∑ Medium-sized mammals		23	24.7	8.7	91.5 ± 29.8	66.7 ± 8.9
	Nyctereutes procyonoides	11	11.8	4.2	118.8 ± 54.1	72.1 ± 12.6
	Unidentified Canidae	1	1.1	0.4	0.4	1.6
	Lepus europaeus	3	3.2	1.1	83.3 ± 7.5	98.2 ± 0.7
	Lepus timidus	1	1.1	0.4	43.0	35.2
	Lepus spp.	7	7.5	2.7	52.0 ± 49.7	45.9 ± 19.2
	Ondatra zibethicus	1	1.1	0.4	142.0	86.9
∑ Large mammals		11	11.8	4.2	37.8 ± 19.1	32.0 ± 13.3
	Alces alces	1	1.1	0.4	180.0	100.0
	Unidentified Cervidae	10	10.8	3.8	23.6 ± 14.1	25.2 ± 12.6
∑ Unidentified mammals		9	9.7	3.4	16.0 ± 15.5	76.5 ± 12.6
∑ Mammals		68	73.1	25.8	50.1 ± 12.5	68.7 ± 4.8
Vol1		93	–	–	71.2 ± 11.0	–
Vol2		93	–	–	81.7 ± 12.6	–
Diversity index		93	–	–	2.8 ± 0.3	–

N = the number of raccoon dog specimens with the observed food item, FO1 = 100×the proportion of stomachs containing each food item, FO2 = 100×the occurrence of each food item/the total number of occurrences of all food items, RS = the volume of each food item of the total volume of the stomach food items, Vol1 = the total volume of all digestible food items excluding baits, Vol2 = the total volume of all ingested material including nondigestible constituents, baits, and groomed raccoon dog hair, Diversity index = the number of different food items per stomach
