# Supplementary material for: Application of change-point analysis to determine winter sleep patterns of the raccoon dog (Nyctereutes procyonoides) from body temperature recordings and a multi-faceted dietary and behavioral study of wintering
Source: BMC Ecol. 2012 Dec 13;12:27. doi: 10.1186/1472-6785-12-27 (PMC3549453; doi:10.1186/1472-6785-12-27)
Supplement: Additional file 3 — Diversity of mammals in the intestines of wild raccoon dogs. [file 1472-6785-12-27-S3.pdf]

Additional file 3. Diversity of mammals in the intestines of wild raccoon dogs.

	N	FO1 (%)	FO2 (%)	Volume (ml)	RS (%)
∑ Small mammals	53	57.0	20.5	9.1 ± 2.2	59.3 ± 5.0
Sorex araneus	1	1.1	0.4	17.5	39.0
Sorex spp.	17	18.3	6.6	6.3 ± 2.0	48.5 ± 8.3
Myodes glareolus	9	9.7	3.5	3.8 ± 1.7	67.0 ± 14.0
Microtus agrestis	9	9.7	3.5	8.4 ± 3.6	52.8 ± 11.6
Microtus spp.	10	10.8	3.9	5.9 ± 2.3	49.0 ± 11.2
Unidentified Arvicolinae	7	7.5	2.7	4.3 ± 1.2	21.5 ± 5.0
Micromys minutus	1	1.1	0.4	22.0	96.1
Apodemus flavicollis	1	1.1	0.4	0.6	65.9
Unidentified Murinae	2	2.2	0.8	14.2 ± 13.9	89.3 ± 10.8
Rattus norvegicus	1	1.1	0.4	100.0	94.0
Sciurus vulgaris	4	4.3	1.5	2.3 ± 1.4	31.6 ± 23.6
∑ Medium-sized mammals	26	28.0	10.0	10.8 ± 2.6	62.3 ± 6.7
Nyctereutes procyonoides	10	10.8	3.9	11.2 ± 2.6	68.6 ± 11.7
Unidentified Mustelidae	2	2.2	0.8	14.2 ± 10.6	72.7 ± 27.2
Lepus timidus	3	3.2	1.2	4.6 ± 0.8	79.7 ± 18.4
Lepus spp.	11	11.8	4.2	11.4 ± 5.5	50.0 ± 9.6
∑ Large mammals	11	11.8	4.2	12.1 ± 5.1	46.8 ± 13.7
Alces alces	1	1.1	0.4	56.0	98.9
Unidentified Cervidae	10	10.8	3.9	7.7 ± 2.8	41.6 ± 14.0
∑ Unidentified mammals	9	9.7	3.5	5.6 ± 1.9	49.4 ± 15.0
∑ Mammals	76	81.7	29.3	12.5 ± 1.9	75.3 ± 3.8
Vol1	93	−	−	21.3 ± 3.2	−
Vol2	93	−	−	25.0 ± 3.3	−
Diversity index	93	−	−	2.8 ± 0.2	−

N = the number of raccoon dog specimens with the observed food item, FO1 = 100×the proportion of intestines containing each food item, FO2 = 100×the occurrence of each food item/the total number of occurrences of all food items, RS = the volume of each food item of the total volume of the intestinal food items, Vol1 = the total volume of all digestible food items excluding baits, Vol2 = the total volume of all ingested material including nondigestible constituents, baits, and groomed raccoon dog hair, Diversity index = the number of different food items per intestine
