# Supplementary material for: Application of change-point analysis to determine winter sleep patterns of the raccoon dog (Nyctereutes procyonoides) from body temperature recordings and a multi-faceted dietary and behavioral study of wintering
Source: BMC Ecol. 2012 Dec 13;12:27. doi: 10.1186/1472-6785-12-27 (PMC3549453; doi:10.1186/1472-6785-12-27)
Supplement: Additional file 4 — Diversity of plants in the stomachs of wild raccoon dogs. [file 1472-6785-12-27-S4.pdf]

Additional file 4. Diversity of plants in the stomachs of wild raccoon dogs.

		N	FO1 (%)	FO2 (%)	Volume (ml)	RS (%)
∑ Crops		30	32.3	11.4	33.9 ± 10.8	32.6 ± 7.2
	Avena sativa	27	29.0	10.2	37.5 ± 11.8	34.4 ± 7.5
	Panicum miliaceum	6	6.5	2.3	0.1 ± <0.01	0.5 ± 0.3
	Hordeum vulgare	1	1.1	0.4	0.7	43.8
	Triticum aestivum	1	1.1	0.4	4.0	2.0
	Sorghum sp.	1	1.1	0.4	0.4	0.2
∑ Berries		20	21.5	7.6	7.2 ± 5.6	8.8 ± 5.5
	Sorbus aucuparia	5	5.4	1.9	24.9 ± 22.1	25.5 ± 15.3
	Vaccinium vitis-idaea	8	8.6	3.0	1.4 ± 1.0	1.9 ± 1.0
	Vaccinium oxycoccos	5	5.4	1.9	0.1 ± 0.1	<0.1 ± <0.1
	Crataegus spp.	2	2.2	0.8	2.6 ± 1.5	15.3 ± 13.1
	Rosa spp.	2	2.2	0.8	0.5 ± 0.4	0.6 ± 0.4
	Aronia sp.	1	1.1	0.4	0.3	0.6
	Amelanchier spicata	1	1.1	0.4	0.4	0.8
	Rubus chamaemorus	1	1.1	0.4	0.2	0.2
	Rubus saxatilis	1	1.1	0.4	0.01	<0.1
	Convallaria majalis	1	1.1	0.4	0.1	0.1
	Unidentified berries	1	1.1	0.4	0.1	0.1
∑ Vegetables		16	17.2	6.1	11.8 ± 4.2	10.3 ± 3.5
	Solanum tuberosum	14	15.1	5.3	12.7 ± 4.4	11.1 ± 3.6
	Daucus carota	7	7.5	2.7	1.2 ± 0.3	0.7 ± 0.2
	Allium cepa	1	1.1	0.4	2.5	4.4
	Vicia faba	1	1.1	0.4	0.2	<0.1
∑ Fruits		7	7.5	2.7	10.2 ± 8.5	6.4 ± 5.0
	Pyrus communis	1	1.1	0.4	60.5	35.6
	Malus domestica	3	3.2	1.1	0.1 ± <0.01	0.1 ± <0.1
	Musa sp.	1	1.1	0.4	9.0	7.4
	Unidentified fruits	3	3.2	1.1	0.4 ± 0.2	0.6 ± 0.4
Other						
	Helianthus annuus	12	12.9	4.5	33.4 ± 14.5	24.9 ± 8.6
	Arachis hypogaea	1	1.1	0.4	51.3	30.2
	Setaria italica	1	1.1	0.4	0.02	<0.1
	Unidentified plants	3	3.2	1.1	0.4 ± 0.2	0.2 ± 0.1
∑ Digestible plants		45	48.4	17.0	41.7 ± 10.1	37.7 ± 6.5
∑ Wild plants		20	21.5	7.6	6.9 ± 5.6	7.3 ± 4.3
∑ Useful plants		44	47.3	16.7	42.5 ± 10.2	37.8 ± 6.4
∑ Undigestible plants		73	78.5	27.7	4.2 ± 1.0	−

N = the number of raccoon dog specimens with the observed food item, FO1 = 100×the proportion of stomachs containing each food item, FO2 = 100×the occurrence of each food item/the total number of occurrences of all food items, RS = the volume of each food item of the total volume of the stomach food items
