# Supplementary material for: Application of change-point analysis to determine winter sleep patterns of the raccoon dog (Nyctereutes procyonoides) from body temperature recordings and a multi-faceted dietary and behavioral study of wintering
Source: BMC Ecol. 2012 Dec 13;12:27. doi: 10.1186/1472-6785-12-27 (PMC3549453; doi:10.1186/1472-6785-12-27)
Supplement: Additional file 5 — Diversity of plants in the intestines of wild raccoon dogs. [file 1472-6785-12-27-S5.pdf]

Additional file 5. Diversity of plants in the intestines of wild raccoon dogs.

		N	FO1 (%)	FO2 (%)	Volume (ml)	RS (%)
∑ Crops		42	45.2	16.2	13.5 ± 5.5	33.2 ± 5.7
	Avena sativa	39	41.9	15.1	14.4 ± 5.9	33.1 ± 5.8
	Panicum miliaceum	5	5.4	1.9	0.1 ± 0.04	20.4 ± 19.9
	Triticum aestivum	1	1.1	0.4	2.3	2.4
	Sorghum sp.	1	1.1	0.4	0.9	0.9
∑ Berries		20	21.5	7.7	2.3 ± 1.3	15.5 ± 7.0
	Sorbus aucuparia	7	7.5	2.7	1.9 ± 1.1	18.4 ± 10.2
	Vaccinium vitis-idaea	4	4.3	1.5	1.6 ± 1.5	13.4 ± 12.1
	Vaccinium oxycoccos	4	4.3	1.5	0.1 ± 0.1	0.5 ± 0.2
	Vaccinium microcarpum	2	2.2	0.8	0.01 ± <0.01	0.2 ± <0.1
	Vaccinium myrtillus	1	1.1	0.4	0.1	0.8
	Crataegus sp.	1	1.1	0.4	8.0	69.6
	Aronia spp.	2	2.2	0.8	6.9 ± 6.8	21.7 ± 21.5
	Amelanchier spicata	1	1.1	0.4	2.5	7.8
	Rubus idaeus	1	1.1	0.4	0.02	0.1
	Polygonatum officinale	1	1.1	0.4	0.1	0.8
	Unidentified berries	2	2.2	0.8	0.3 ± 0.2	1.0 ± 0.5
∑ Vegetables		13	14.0	5.0	2.5 ± 1.3	5.9 ± 1.9
	Solanum tuberosum	13	14.0	5.0	2.4 ± 1.3	5.8 ± 1.9
	Daucus carota	3	3.2	1.2	0.3 ± 0.2	0.5 ± 0.3
∑ Fruits		9	9.7	3.5	1.5 ± 0.5	13.2 ± 10.3
	Pyrus communis	4	4.3	1.5	1.3 ± 0.9	3.4 ± 2.8
	Malus domestica	2	2.2	0.8	0.1 ± <0.01	0.2 ± <0.1
	Musa sp.	2	2.2	0.8	1.9 ± 1.2	3.8 ± 2.9
	Unidentified fruits	2	2.2	0.8	2.2 ± 1.3	48.7 ± 45.9
Other						
	Helianthus annuus	13	14.0	5.0	15.8 ± 7.3	22.6 ± 7.6
	Arachis hypogaea	1	1.1	0.4	9.5	27.6
	Setaria italica	1	1.1	0.4	0.1	2.1
	Unidentified nuts	1	1.1	0.4	0.1	2.7
	Unidentified plants	6	6.5	2.3	1.8 ± 1.5	10.1 ± 6.1
∑ Digestible plants		61	65.6	23.6	14.5 ± 4.3	37.5 ± 5.1
∑ Wild plants		18	19.4	6.9	1.3 ± 0.7	10.8 ± 5.0
∑ Useful plants		60	64.5	23.2	14.4 ± 4.4	35.8 ± 5.1
∑ Undigestible plants		85	91.4	32.8	2.4 ± 0.4	−

N = the number of raccoon dog specimens with the observed food item, FO1 = 100×the proportion of intestines containing each food item, FO2 = 100×the occurrence of each food item/the total number of occurrences of all food items, RS = the volume of each food item of the total volume of the intestinal food items
