# Supplementary material for: Application of change-point analysis to determine winter sleep patterns of the raccoon dog (Nyctereutes procyonoides) from body temperature recordings and a multi-faceted dietary and behavioral study of wintering
Source: BMC Ecol. 2012 Dec 13;12:27. doi: 10.1186/1472-6785-12-27 (PMC3549453; doi:10.1186/1472-6785-12-27)
Supplement: Additional file 6 — Diversity of birds in the stomachs and intestines of wild raccoon dogs. [file 1472-6785-12-27-S6.pdf]

Additional file 6. Diversity of birds in the stomachs and intestines of wild raccoon dogs.

		N	FO1 (%)	FO2 (%)	Volume (ml)	RS (%)
Stomach	Phasianidae					
	Tetrastes bonasia	2	2.2	0.8	3.1 ± 1.1	2.3 ± 1.1
	Lyrurus tetrix	1	1.1	0.4	92.0	86.8
	Corvidae					
	Garrulus glandarius	1	1.1	0.4	109.0	92.8
	Pica pica	1	1.1	0.4	10.0	76.3
	Strigidae					
	Asio otus	1	1.1	0.4	214.7	92.8
	Paridae					
	Parus caeruleus	1	1.1	0.4	18.5	13.5
	Anatidae					
	Anas sp.	1	1.1	0.4	33.0	98.2
	Laridae					
	Larus sp.	1	1.1	0.4	86.0	63.6
	Unidentified waterfowl	1	1.1	0.4	376.0	99.9
	Unidentified shorebirds	1	1.1	0.4	0.1	0.1
	Other unidentified birds	14	15.1	5.3	0.8 ± 0.4	23.5 ± 9.0
	∑ Birds	25	26.9	9.5	38.2 ± 17.3	38.3 ± 8.2
Intestine	Phasianidae					
	Tetrastes bonasia	1	1.1	0.4	0.3	1.7
	Corvidae					
	Garrulus glandarius	1	1.1	0.4	6.0	49.6
	Anatidae					
	Anas sp.	1	1.1	0.4	25.2	100.0
	Laridae					
	Larus sp.	1	1.1	0.4	42.0	47.7
	Unidentified waterfowl	1	1.1	0.4	33.0	100.0
	Other unidentified birds	6	6.5	2.3	2.8 ± 1.5	25.5 ± 16.7
	∑ Birds	11	11.8	4.2	11.2 ± 4.5	41.1 ± 12.9

N = the number of raccoon dog specimens with the observed food item, FO1 = 100×the proportion of stomachs/intestines containing each food item, FO2 = 100×the occurrence of each food item/the total number of occurrences of all food items, RS = the volume of each food item of the total volume of the stomach/intestinal food items
