# Supplementary material for: Application of change-point analysis to determine winter sleep patterns of the raccoon dog (Nyctereutes procyonoides) from body temperature recordings and a multi-faceted dietary and behavioral study of wintering
Source: BMC Ecol. 2012 Dec 13;12:27. doi: 10.1186/1472-6785-12-27 (PMC3549453; doi:10.1186/1472-6785-12-27)
Supplement: Additional file 7 — Diversity of fish in the stomachs and intestines of wild raccoon dogs. [file 1472-6785-12-27-S7.pdf]

Additional file 7. Diversity of fish in the stomachs and intestines of wild raccoon dogs.

		N	FO1 (%)	FO2 (%)	Volume (ml)	RS (%)
Stomach	Percidae	7	7.5	2.7	23.0 ± 8.8	25.6 ± 8.7
	Cyprinidae	2	2.2	0.8	25.1 ± 15.6	18.1 ± 12.3
	Abramis brama	1	1.1	0.4	108.0	100.0
	Esocidae					
	Esox lucius	2	2.2	0.8	21.5 ± 15.5	23.9 ± 20.3
	Lotidae					
	Lota lota	1	1.1	0.4	1.4	0.7
	Unidentified fish	9	9.7	3.4	1.5 ± 1.0	4.5 ± 2.5
	∑ Fish	19	20.4	7.2	19.9 ± 7.8	21.3 ± 7.4
Intestine	Percidae	3	3.2	1.2	2.5 ± 2.0	17.5 ± 13.8
	Cyprinidae	1	1.1	0.4	0.1	0.7
	Abramis brama	1	1.1	0.4	6.0	100.0
	Esocidae					
	Esox lucius	1	1.1	0.4	0.2	0.6
	Unidentified fish	4	4.3	1.5	3.6 ± 1.6	20.4 ± 9.4
	∑ Fish	10	10.8	3.9	2.8 ± 0.9	23.5 ± 10.1

N = the number of raccoon dog specimens with the observed food item, FO1 = 100×the proportion of stomachs/intestines containing each food item, FO2 = 100×the occurrence of each food item/the total number of occurrences of all food items, RS = the volume of each food item of the total volume of the stomach/intestinal food items
