# Supplementary material for: Application of change-point analysis to determine winter sleep patterns of the raccoon dog (Nyctereutes procyonoides) from body temperature recordings and a multi-faceted dietary and behavioral study of wintering
Source: BMC Ecol. 2012 Dec 13;12:27. doi: 10.1186/1472-6785-12-27 (PMC3549453; doi:10.1186/1472-6785-12-27)
Supplement: Additional file 8 — Diversity of invertebrates in the stomachs and intestines of wild raccoon dogs. [file 1472-6785-12-27-S8.pdf]

Additional file 8. Diversity of invertebrates in the stomachs and intestines of wild raccoon dogs.

		N	FO1 (%)	FO2 (%)	Volume (ml)	RS (%)
Stomach	Hymenoptera					
	Braconidae	1	1.1	0.4	0.1	0.07
	Vespula sp.	1	1.1	0.4	0.1	0.07
	Bombus sp.	1	1.1	0.4	0.6	1.8
	Diptera					
	Calliphoridae	1	1.1	0.4	0.1	0.2
	Fanniidae	1	1.1	0.4	0.1	0.2
	Coleoptera					
	Chrysomelidae	1	1.1	0.4	0.1	20.0
	Geotrupes sp.	1	1.1	0.4	0.1	20.0
	Melolontha hippocastani	1	1.1	0.4	0.1	20.0
	Siphonaptera	1	1.1	0.4	≤0.01	9.1
	∑ Insects	7	7.5	2.7	≤0.20 ± 0.07	10.2 ± 8.4
	Lumbricidae	1	1.1	0.4	0.1	0.06
	∑ Invertebrates	8	8.6	3.0	≤0.19 ± 0.07	8.9 ± 7.4
Intestine	Hymenoptera					
	Apocrita	1	1.1	0.4	≤0.01	100.0
	Vespula sp.	1	1.1	0.4	≤0.05	0.15
	Diptera					
	Calliphoridae	1	1.1	0.4	0.1	0.3
	Coleoptera					
	Geotrupes sp.	1	1.1	0.4	0.1	0.7
	Siphonaptera	2	2.2	0.8	≤0.01 ± <0.001	0.7 ± 0.4
	Hemiptera					
	Heteroptera	2	2.2	0.8	≤0.08 ± 0.03	0.3 ± 0.1
	Psylloidea	1	1.1	0.4	≤0.01	0.04
	Mallophaga	1	1.1	0.4	≤0.01	1.5
	∑ Insects/Invertebrates	9	9.7	3.5	≤0.05 ± 0.02	11.6 ± 11.0

N = the number of raccoon dog specimens with the observed food item, FO1 = 100×the proportion of stomachs/intestines containing each food item, FO2 = 100×the occurrence of each food item/the total number of occurrences of all food items, RS = the volume of each food item of the total volume of the stomach/intestinal food items
