# Supplementary material for: Application of change-point analysis to determine winter sleep patterns of the raccoon dog (Nyctereutes procyonoides) from body temperature recordings and a multi-faceted dietary and behavioral study of wintering
Source: BMC Ecol. 2012 Dec 13;12:27. doi: 10.1186/1472-6785-12-27 (PMC3549453; doi:10.1186/1472-6785-12-27)

Additional file 9. Distribution of dietary items in the stomachs and intestines of the raccoon dogs. (a) Main categories, **(b)** categories of mammals, **(c)** categories of digestible plant material; plots depict the FO1 of the categories as the percent of the total chart area (n = 93).

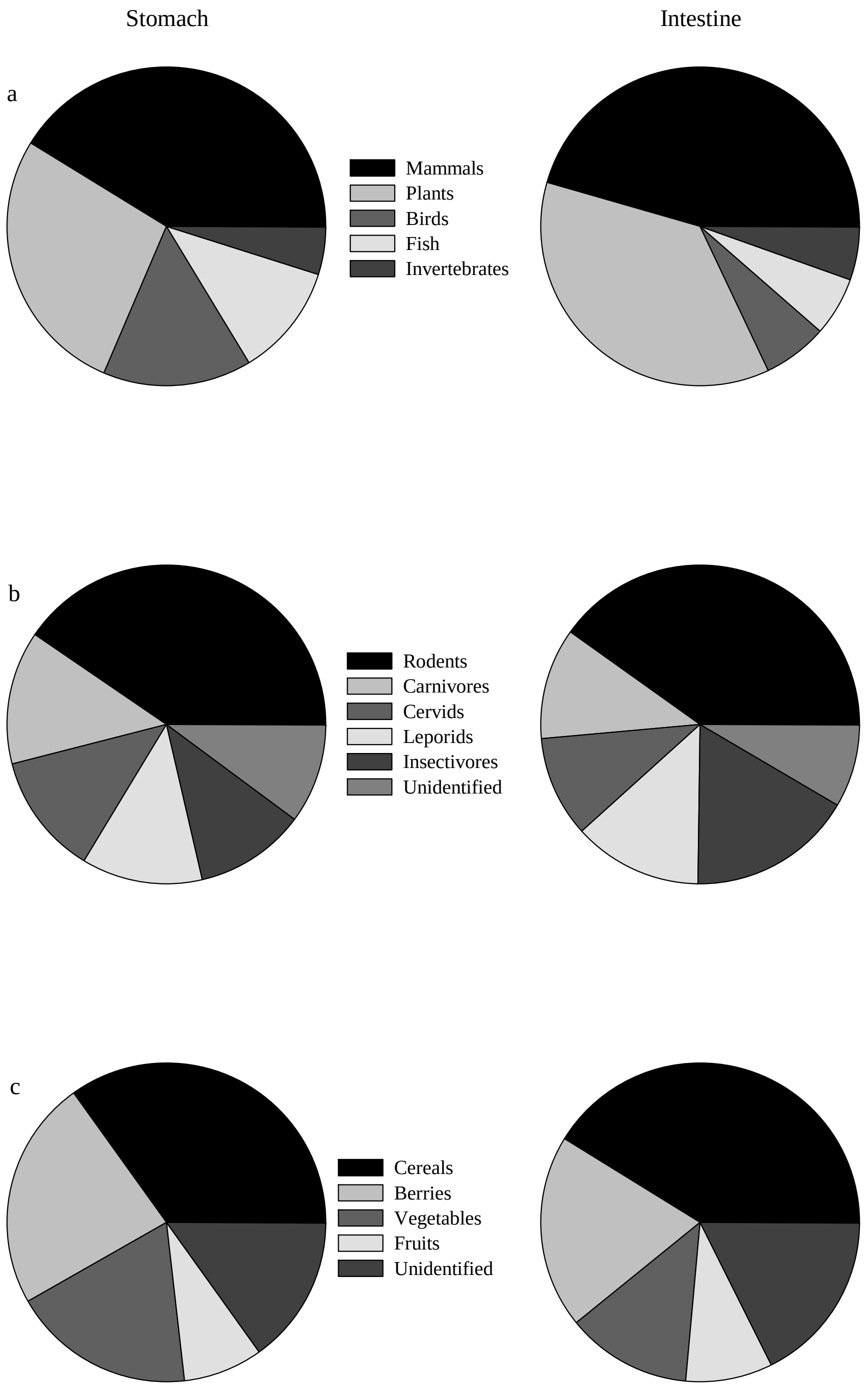

Supplement: Additional file 9 — Distribution of dietary items in the stomachs and intestines of wild raccoon dogs. [file 1472-6785-12-27-S9.pdf]
