# Supplementary material for: Application of change-point analysis to determine winter sleep patterns of the raccoon dog (Nyctereutes procyonoides) from body temperature recordings and a multi-faceted dietary and behavioral study of wintering
Source: BMC Ecol. 2012 Dec 13;12:27. doi: 10.1186/1472-6785-12-27 (PMC3549453; doi:10.1186/1472-6785-12-27)

Additional file 10. Relative changes (%) in the proportions of selected adipose tissue fatty acids (FA) during winter. The FA were measured from subcutaneous white adipose tissue of wild raccoon dogs. + values indicate that a FA increased in proportion and – values signify its decrease in proportion during overwintering (mean + SE; n = 14).

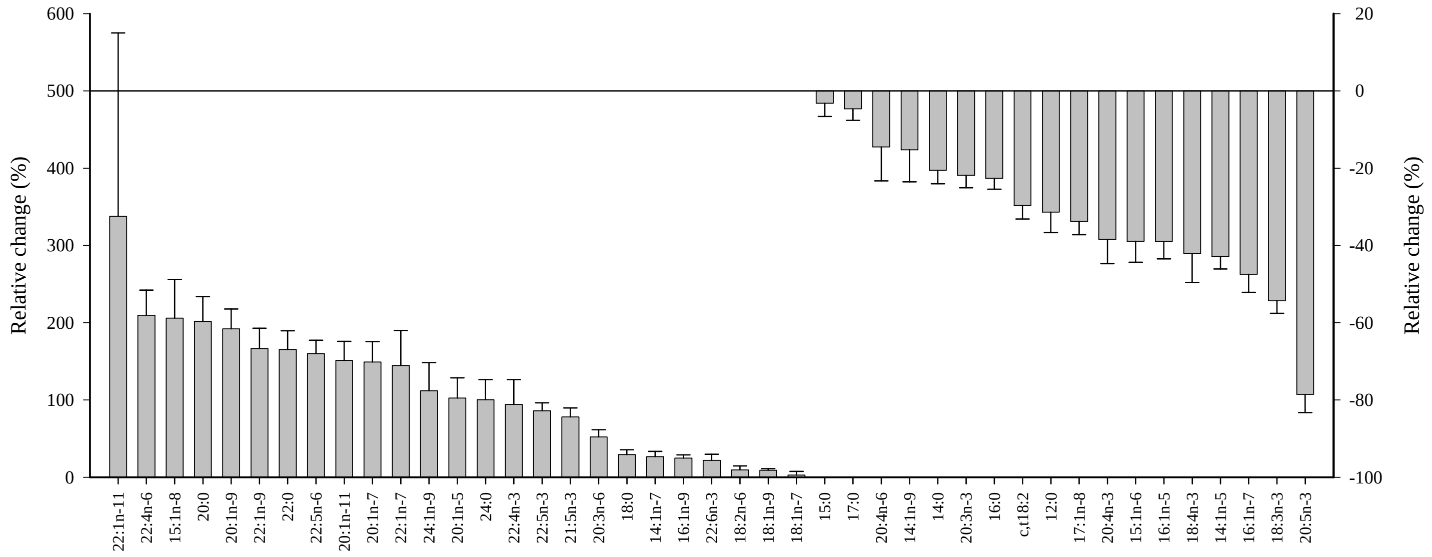

Supplement: Additional file 10 — Relative changes in the proportions of selected adipose tissue fatty acids during winter in wild raccoon dogs. [file 1472-6785-12-27-S10.pdf]
